# Release of coarse woody detritus-related carbon: a synthesis across forest biomes

**DOI:** 10.1186/s13021-019-0136-6

**Published:** 2020-01-15

**Authors:** Mark E. Harmon, Becky G. Fasth, Misha Yatskov, Douglas Kastendick, Joachim Rock, Christopher W. Woodall

**Affiliations:** 1 Department of Forest Ecosystems and Society, Oregon State University, 321 Richardson Hall, Corvallis, OR 97331 USA; 2 Department of Earth, Ocean, and Atmospheric Science, Oregon State University, Corvallis, OR 97331 USA; 3 USDA Forest Service, PNW Research Station, Anchorage Forestry Sciences Lab, 161 E 1st Ave., Door 8, Anchorage, AK 99501 USA; 4 Northern Forest Science and Applications, USDA Forest Service Northern Research Station, 271 Mast Road, Durham, NH 03824-0640 USA; 5 Thünen Institute of Forest Ecosystems, Alfred-Möller-Str. 1, 16225 Eberswalde, Germany

**Keywords:** Coarse woody detritus, Decomposition, Forest carbon balance, Forest disturbance consequences, Heterotrophic respiration, Tree mortality effects

## Abstract

**Background:**

Recent increases in forest tree mortality should increase the abundance coarse woody detritus (CWD) and ultimately lead to increased atmospheric carbon dioxide. However, the time course of carbon release from CWD is not well understood. We compiled CWD decomposition rate-constants (i.e., *k*) to examine how tree species, piece diameter, position (i.e., standing versus downed), canopy openness, and macroclimate influenced *k*. To illustrate their implications we modeled the effect of species and position on estimates of decomposition-related carbon flux. We examined a subset of currently used models to determine if their structure accounted for these factors.

**Results:**

Globally *k* of downed CWD varied at least 244-fold with interspecies variation at individual sites up to 76-fold. While *k* generally decreased with increasing piece diameter, under open canopies the opposite occurred. Standing CWD sometimes exhibited little decomposition, but sometimes had *k* values up to 3 times faster than downed CWD. There was a clear response of *k* to mean annual temperature of ≈ 2.6 times per 10 ℃; however, there was considerable variation for a given mean annual temperature related to species, diameter, and position. A key feature of carbon release from CWD after disturbance was the “evolution” of the ecosystem-level *k* value as positions and species mixtures of the remaining CWD changed. Variations in decomposition caused by disturbance (e.g., changes in species, positions, sizes, and microclimate) had the potential to cause net carbon fluxes to the atmosphere to be highly nonlinear. While several models currently being used for carbon accounting and assessing land-use/climate change would potentially capture some of these post disturbance changes in fluxes and carbon balances, many would not.

**Conclusions:**

While much has been learned in the last 5 decades about CWD decomposition, to fully understand the time course of carbon release from increased mortality and other aspects of global change a new phase of global CWD research that is more systematic, experimental, and replicated needs to be initiated. If our findings are to be fully applied in modeling, an approach acknowledging how the rate of carbon release evolves over time should be implemented.

## Background

A predicted response to a warming global climate is an increase in major forest ecosystem disturbances such as those caused by hurricanes, fire, drought, and insects [1–3]. Consequently, in the last decade, considerable effort has been made to estimate the amount of mortality related to forest disturbance particularly in temperate and boreal regions (e.g., [4]. For example, recent outbreaks of bark beetles have killed trees on 6 to 11 million ha in North America in a little over a decade [5]. Fire incidence and severity has been observed to increase in the western US as well as Amazonia in response to climatic drying [6, 7]. Widespread droughts have also led to a major tree die off in the Southwestern US [2, 8], western Canada [9, 10], and southern Europe [11]. While it is not completely certain, hurricane incidence and intensity appear to have both increased; these storms can kill a considerable amount of live tree biomass with hurricanes Katrina and Rita creating 43.9 and 37.9 Tg C of woody detritus, respectively [12]. The resulting influx of detritus can affect stand development processes for decades in some locations [13]. However, a changing climate is not the only factor leading to recent increases in tree mortality: Invasions of diseases such as sudden oak disease (*Phytophthora ramorum)* [14] and insects such as the emerald ash borer (*Agrilus planipennis*) [15, 16] and hemlock woolly adelgid (*Adelges tsugae*) [17] are causing wide spread mortality of their new host tree species. Moreover, there is increasing evidence that small scale mortality (i.e., so-called regular or background mortality) has been increasing in western North America [18, 19] and southern Europe [11]. In addition to mortality events, if rates of stand development accelerate in response to climate change [20], one would expect greater density-induced tree mortality with concomitant dead wood creation. Taken together, these studies indicate considerable amounts of woody carbon are being transferred from live to dead pools by these various causes of mortality and that this process is likely to increase in the future.

In addition to “natural” disturbances, management-related disturbances can also create woody detritus. Removal of woody detritus after either type of disturbance can have major impacts on forest carbon stores as well as the timing of carbon emissions following disturbance. Compared to natural disturbances, management activities such as salvage harvest have the potential to remove a higher proportion of disturbance generated woody detritus [21]. As salvaged wood is often converted into products (e.g., paper and energy) with a shorter life-span than woody detritus, the general impact of salvage harvest is to reduce carbon stores [22–24] and speed the release of carbon to the atmosphere [25, 26]. The degree to which the stores and rate of carbon release are impacted depends on the differences between natural decay-related carbon release versus release from forest product carbon emissions; although we note that because of the paucity of data these life-spans are often assumed to be the same [26]. Given the potential variation in the rate of woody detritus decomposition [21, 27], the specific carbon policy implications of salvage for products and/or fuel reduction are likely to vary widely. Hence, more fully understanding the factors controlling decomposition of woody detritus is important to developing a science-based carbon management policy for forests.

Since the time course of mortality-related carbon release is not well understood, two simplifying assumptions are commonly used, both of which equate mortality with carbon release. The first is to assign mortality to a “committed emission” that eventually will be released (e.g., IPCC Tier 1 guidelines [28]). While undoubtedly true, the influence of mortality on the carbon balance depends on the time course of emissions versus uptake by the ecosystem. The second is to assume the system is in steady-state, which implies that the amount being added to the dead pools via mortality is equivalent to the amount being lost to the atmosphere (e.g., [29]). However, since the rate of this disturbance-related mortality appears to be increasing over broad regions, the steady-state assumption is not valid in many cases. Estimating the effect of mortality, therefore, requires one to examine the transient response which is in turn dependent on the decomposition rate of dead wood generated by disturbance-related mortality. The same is true for fully understanding the impact of management actions such as salvage, fuel reduction, and biomass energy production. Moreover, since carbon released earlier to the atmosphere has more potential to influence climate than later [30], the specific timing of decomposition-related carbon emissions is important.

While decomposition is a fundamental ecological process, it is not well understood relative to other processes such as production [21]. Decomposition losses can be divided into those associated with respiration, fragmentation, and leaching [31]. While respiration represents a transfer to the atmosphere, fragmentation and leaching represent transfers within the ecosystem and a change to a smaller size or dissolved form, respectively. The proportion of carbon flowing through these sub-processes is not well quantified, although it is often assumed that respiration comprises the majority. Though it is possible to quantify these processes [32, 33], most studies examining the decomposition of woody detritus quantify the changes of overall mass, density (i.e., mass/volume), and/or volume. To estimate the overall rate of decomposition a single negative exponential model has widely been used:

1 $${\text{M}}_{{\text{t}}} = {\text{M}}_{0} {\text{e}}^{{ - k{\text{t}}}}$$

where M is the metric of choice (mass, volume, or density), t is time, and *k* is the rate-constant of decomposition [27]. Although other models have also been used to describe the course of decomposition to capture the heterogeneous nature of woody detritus or time lags associated with decomposer colonization [27, 34], the use of the single negative exponential model and the rate constant *k* has been widespread as it provides a simple, integrated index of this complex process.

Our objective is to provide insight into the nature of transient emission of carbon from coarse woody debris due to disturbances to refine our understanding of how recent increases in tree mortality could affect the global carbon balance. To achieve this we first review what is currently known about the decomposition of coarse woody detritus (CWD) in boreal, temperate and tropical forests. CWD, defined here as dead woody material at least 1 m long and greater than 10 cm diameter at the thinner end, is the largest share of the dead wood carbon pool generated by major disturbances. Thus, while disturbance also generates dead branches as well as dead coarse roots, our analysis focuses on the larger-sized wood. Our review of available data is based on published and unpublished rates of CWD decomposition that we have gathered over the course of several decades. The decomposition of dead organic material involves mechanical and chemical processes and depends on decomposer community structure, their development and succession over time, and all the factors influencing their activity ([35–38]. Since information on this detailed level across the breadth of global sampling is very scarce, we examined general factors that influence the rate at which CWD decomposes including tree species, size, position (i.e., standing versus downed), canopy openness, and climate. Finally, we explore the implications of observed decomposition rates on modeling carbon release, examine a range of current modeling approaches, and make suggestions for the next steps in quantifying and modeling this important process.

## Data sources and methods

The estimates of CWD decomposition rates used in our analysis were based on both published and unpublished data. For published studies of decomposition rates we selected those based for the most part on individual pieces of CWD. Therefore, studies that estimated decomposition rate solely based on inventories of stores/stocks (e.g., [39]) or models (e.g., [40–43] were not included. As an exception, we used estimates based on the ratio of mortality and an assumption that the observed store was in steady-state. For unpublished data we used our own databases which have been gathered over the course of several decades. We provide summary estimates based on these data for the many species and locations we have examined. The course of decomposition can be described using volume, mass, or density loss of a piece of CWD; moreover, different equation types can be used to determine a rate of loss. We analyzed all *k* values found, because the rate-constants calculated from these different combinations differ less than between studies (*ceteris paribus*) or species. The influence of the different factors analyzed on *k* is also irrespective of the way the rate-constant was estimated. As the comparison for all the factors except climate was within a study, this method would not have influenced the relative differences. For climate, we acknowledge that this method may have somewhat reduced the proportion of variance explained by the climatic variable examined.

There are three basic methods for determining decomposition rates of individual CWD pieces and each may give a different estimate: (A) Chronosequences are the most common and involve a substitution of space for time. While this method can span a considerable time period (i.e., hundreds of years in some cases), there are uncertainties related to the initial condition of the pieces, the dating of mortality, and the uniformity of environmental conditions. (B) Time series (i.e., following individual pieces over time) is an ideal method in terms of knowing the date of mortality and the initial condition of the pieces. These studies typically are short term relative to the lifespan of CWD; typically lasting less than 30 years. C) Decomposition vectors combine aspects of chronosequences and time series by sampling a wide range of ages (or in some cases decay stages) over two points in time [44]. Since the pieces are resampled, the rate of change over time is more certain than for a chronosequence, and since a wide range of states is examined the long-term trend is better represented than for the typical time series. However, since the decomposition vectors for resampled pieces do not estimate an overall rate of change for a species, estimates from the various stages have to be averaged, usually by weighting by the relative time in each stage or state that is examined. This provides estimates similar to chronosequences and time series, but there is uncertainty in terms of the residence time of each stage. Finally, for a population of dead wood pieces one may estimate decomposition rate-constants from the ratio of dead wood input to stores. This assumes that the dead wood store has reached steady-state and is typically applied to forests which have not been subject to major mortality events for considerable time periods.

In addition to compiling data on decomposition-rate constants for CWD, we also used site description data when provided by the articles. Climate was characterized by mean annual temperature (MAT) and average total annual precipitation (TAP). In many cases temperature was described as a range (typically the average for the coldest and warmest months), which we averaged to approximate the MAT. When more than one site was used and the decomposition data were reported by site we used the site-specific climate data. Otherwise we averaged climatic data for the sites. For articles that did not provide climatic data, but the location was described adequately, we used the location to search online for climate data to approximate temperature and precipitation typically from a nearby meteorological station. However, there were some studies in which the location was too vague to do this and they were excluded from the analyses involving climate. To provide a more consistent estimate of precipitation and temperature we also used the location given by the studies and the WorldClim2 database [45] at a 2.5 min resolution to determine monthly values that were then either summed or averaged to give TAP and MAT, respectively.

Although other studies have used involved statistical analysis such as linear mixed modeling to derive relationships between variables such as species, size, and climate versus decomposition rate-constants (e.g., [40–42]), we feel the overall sampling design with respect to the factors we examined is severely unbalanced, that differences in field and laboratory methods influence the results of analyses and that these statistical methods, despite their high level of sophistication, are thus inappropriate. For example, while using meta-analysis would be desirable, it requires a degree of randomization not present in most of the studies we analyzed. Moreover, even computation of a regional or global average and dispersion statistics would be misleading given the subjective nature of the overall sampling design. We therefore used alternative methods to document the effect of the factors of interest. We first noted whether a factor had been examined and an effect had been reported. The next step was to quantify the sign and magnitude of the effect. In the case of the species effect we used the ratio of the fastest to the slowest decomposition-rate constant. For position effects, we used the ratio of standing dead to downed dead decomposition rate-constant as the index. For size, we only noted the sign of the effect given that range of sizes examined was so variable that a comparison of magnitude could be misleading. For the comparison of open- versus closed-canopy sites (i.e., those with and without overstory trees) we also noted the sign of the effect in part because there were few studies that actually made this comparison and hence an average magnitude would be too uncertain.

In the case of three variables, initial wood density (IWD), MAT, and TAP, it was possible to use regression analysis to assess an effect; however, even here caution must be exercised in interpretation as many other factors (e.g., species decay resistance, size, and position) could be confounded with these variables. For IWD statistical models we used linear regression (proc GLM, [46]) for the *k* estimates with associated IWD to determine the relationship for the entire dataset as well as for each major forest biome (i.e., boreal, temperate, tropical). In the case of TAP we used linear and polynomial regression (proc GLM, [46]) to assess a relationship with *k* and did not examine the effect of seasonality. For MAT we used non-linear regression (proc NLIN, [46]) to determine the Q10 (Quotient 10) for the dataset as a whole, standing versus downed CWD, and species with low decay resistance versus those with moderate to high decay resistance. While there are other models of temperature response, Q10 is perhaps the simplest in that it determines the increase in a biological process with a 10 C increase in temperature. Analysis of the climate variables was done for the values reported, those indicated by WorldClim2, and for a hybrid set of values that replaced WorldClim2 precipitation predictions when local estimates with high levels of confidence were available.

After synthesizing what is known about decomposition rates of CWD and its controls across global forest biomes we explored their implications for modeling carbon release. In particular we used a sensitivity analysis to explore the potential effects of species differences and the transfer of CWD from one position (standing) to another (downed). While theoretical, this analysis was guided by the differences observed in the dataset between species and positions. Two aspects of species effects were examined: the difference among species in terms of 1) *k* and 2) abundance. We modelled decomposition for a total of 10 “species” with a ratio of fastest to slowest *k* ranging between either 1, 2, 4, 8, or 16. The first ratio indicated all species had identical *k*’s, whereas the latter indicated up to a 16-fold difference among species. For all simulations the unweighted average *k* was kept at 0.05 per year and species *k*’s were assigned at equal intervals (i.e., 0.1 the difference between the fastest and slowest *k;* Additional file 1: Figure S4). Five different abundance patterns of species were assessed: (1) uniform (i.e., all the species contributed the same amount of input), (2) a normal distribution in which the species with average k’s made the greatest contribution, (3) bimodal distribution in which fastest and slowest decomposing species made the greatest contribution, (4) a “slow” biased distribution in which slowest decomposing species made the greatest contribution and (5) a “fast” biased distribution in which the fastest decomposing species made the greatest contribution (Additional file 1: Figure S5). To assess the effects of variation in *k* and species abundance we used the relative difference in carbon flux between a particular case (e.g., twofold difference in maximum and minimum *k* and uniform distribution) and the reference case in which all species had the same *k*.

To assess the impact changes in microenvironment associated with changes in position we modeled the common situation in which a disturbance created standing dead trees (i.e., snags) and these snags fall over time. Snag fall rate was simulated by assuming snags either started to fall immediately or needed a 10-year period for snag fall to begin. Once snags began to fall three time frames for the majority of snags to fall were explored: 15 years, 30 years, and 60 years. It was assumed that the *k* of maximum decomposition was 0.05 per year. In one case, representing the situation in mesic to arid climates, it was assumed that snags would decompose slower than logs (i.e., downed wood) and the ratio of snag to log decomposition *k*’s was either 1, 0.5, 0.25, 0.125, or 0. In the other case it was assumed that snags would decompose faster than logs and the log to snag decomposition *k* ratio was either 1, 0.5, 0.25, 0.125, or 0. The latter case represents a common situation in boreal forests in which the soil is saturated and mosses quickly over top logs [47]. To assess the effect of transitions between positions, we quantified the timing and magnitude of the maximum carbon flux.

Our analysis focused primarily on the release of carbon from CWD. However, to understand the impact of this release on the overall ecosystem carbon balance one has to also consider net uptake via net primary production (NPP). We present some preliminary, but useful examples by using a relatively simple model to examine the effects of mortality on forest ecosystem carbon balance as indicated by net ecosystem production (NEP). There were two pools, live and dead, that represented the store of wood carbon at the landscape to regional level (Additional file 1: Figure S11). The NPP base-level was assumed to be 2 MgC/ha/year, which represented an average over succession. The mortality base-level was assumed to be 0.01 per year, which corresponded to a maximum tree life-span (at least for 99% of the trees) of 460 years. Three base-levels of decomposition rate-constants were used: 0.025, 0.05, and 0.1 per year to correspond the majority of observed values in boreal and temperate forests. NEP was calculated as NPP minus decomposition-related losses which were assumed to be primarily from respiration.

Net changes in live stores were modeled as NPP minus mortality losses. The latter was calculated from the mortality rate-constant and the live store in the previous time step. Net changes in dead stores were modeled as the mortality losses from live stores minus the decomposition loss. The latter was calculated from the decomposition rate-constant and the dead store in the previous time step. NPP, mortality, and decomposition were altered using a step function (smoothed by a 20 year window) that indicated the duration and magnitude of the change (Additional file 1: Figure S12).

To provide a sense of how CWD-related carbon is modeled in global change-related analyzes, we conducted a short review of how this pool is treated in forest and vegetation models currently used (e.g., in carbon accounting under either the Kyoto Protocol or European Union legislation [48]). We also looked at models used in in the “Inter-Sectoral Impact Model Intercomparison Project” (ISIMIP) “forests” and “biomes” sectors. The full list of these models can be found at [49]. ISIMIP is a scientific community-driven “framework for consistently projecting the impacts of climate change across affected sectors and spatial scales” aimed at improving information about possible futures by delivering comparable projections based on different models, but identical input data [49]. Results derived from the models we reviewed have been cited in the previous IPCC Assessment Reports, and results from current efforts using the models are very likely to be cited in the next Assessment Report (AR 6). Therefore, we were interested in how decomposition of woody detritus was treated in these models and we used the information presented or cited on the respective models’ ISIMIP web page [49] available 18th December 2019. Based on the structure of these models (e.g., highly aggregated soil-based models to many CWD-related pools), we considered the degree to which models could mimic the variation in decomposition rates, temporal fluxes, and balances of carbon in disturbed forests we found in our analysis.

## Results and discussion

### General description of studies

#### Location of estimates

**Fig. 1:**
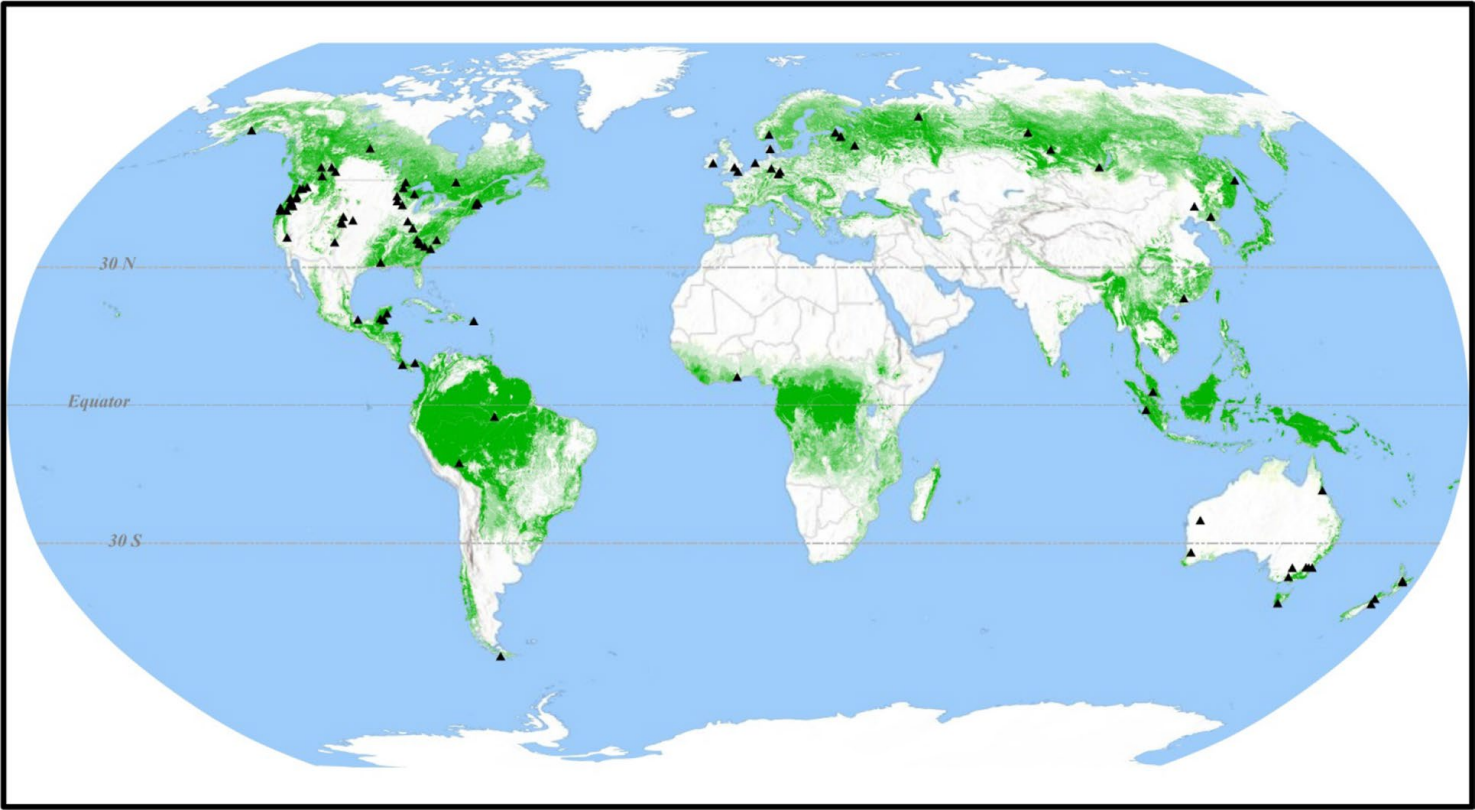
Location of studies that have determined coarse woody detritus decomposition rates (forests indicated by green shaded land areas)

Geographically the majority of estimates (61%) have been in temperate forests, with the highest concentration in North America (Fig. 1, Additional file 2: Table S-1). Estimates from boreal forests comprised 25% of the total, the largest share of these were from Scandinavia and northwestern Russia. Despite some of the earliest estimates of CWD decomposition rates having been made in tropical forests [50], very few estimates have since come from tropical forests (14%). This lack of estimates may be in part due to the aggregation of species. Another notable geographic pattern was the lack of data from the southern hemisphere, although Australia and New Zealand represent a notable area of concentrated work in that hemisphere.

#### Climatic conditions examined

**Fig. 2:**
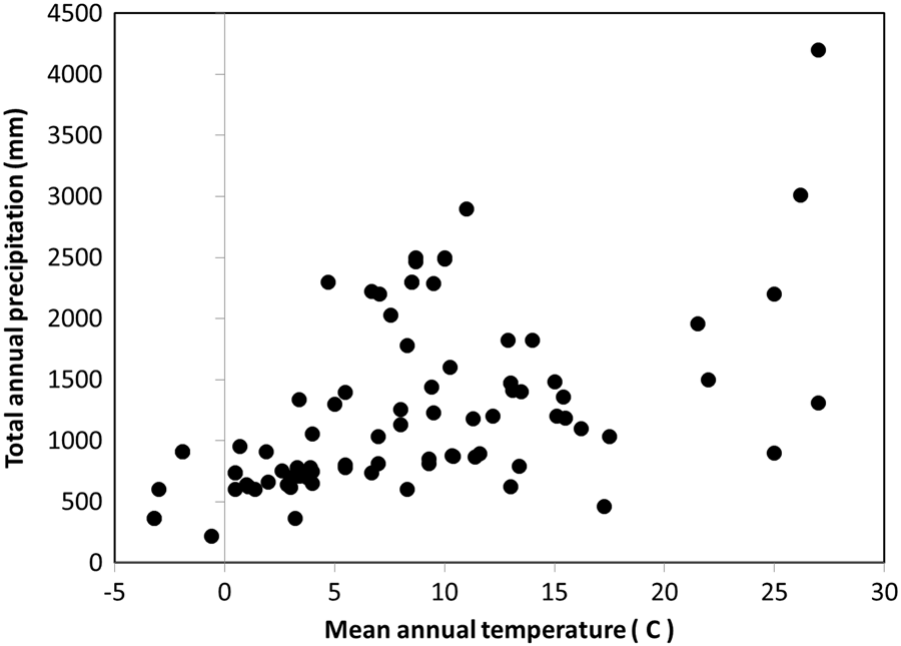
Climatic conditions of decomposition studies for coarse woody detritus based on climate reported by studies

The climatic conditions in the studies examined in our synthesis were quite broad (Fig. 2). Based on the values reported in studies, MAT ranged from − 3.2 to 27 ℃ and TAP ranged from 216 to 4200 mm per year. For the WorldClim2 database MAT ranged from − 1.81 to 26.8 ℃ and TAP ranged from 490 to 4058 mm per year. Although there was a strong relationship between the climate reported by studies and WorldClim2, some notable exceptions existed particularly for precipitation in mountainous regions (see Additional file 1). While many regions of climatic space appeared well sampled, there was a sparsity of data from subtropical (17 to 22 ℃) as well as warm, moist sites (> 1500 mm TAP). In addition, temperate-arid woodlands such as those in the southwestern US were poorly represented, although this region has also been subject to considerable recent disturbance [8].

#### Estimation method and uncertainty

A total of 295 estimates of CWD decomposition rate-constants (i.e., *k*) from 78 different data sources were reported (Additional file 2: Table S-1). Of these, 71% were chronosequence-based, 20% were time series-based, 5% were decomposition vector-based, 3% were based on steady-state estimates, and 1% relied on a mix of methods. Change in density (48% of cases) and mass (50% of estimates) were used most to determine decomposition rates and volume the least (< 1% of cases). For roughly one-third of the estimates the standard error of *k* was reported, with an average relative value of 23%. There was considerable range in this index of *k* estimate variability: the lowest was 1% and the highest 300%. Seven (6%) of the estimates had a relative standard error of over 50%, either because of low values of *k* (4 cases) or low sample size (3 cases). The vast majority of estimates (78 cases or 70%) of *k* had a relative standard error of less than 20%. Moreover, a large share (46 cases or 40%) had relative uncertainty less than 10%. This suggests that when adequately sampled *k* differences of more than 20% could be putatively viewed as significantly different.

### Range of estimates

The slowest rate of decomposition occurred in snags of conifer species, with a *k* of effectively zero, but for some species (e.g., *Eucalyptus camaldulensis*) downed pieces also had very slow rates of decomposition (0.004 per year) [51]. The fastest rate of decomposition (0.975 per year) was estimated for the tropical species *Heliocarpus appendiculatus* (Malvaceae) [52] (Additional file 2: Table S-1). This data suggest that downed CWD decomposition rates can globally vary at least 244-fold. Expressed as the time required to decompose 95% of the initial mass, these data indicate downed CWD could last as short as 3 years or as long as 750 years. The longevity based on these studies may be underestimated given that well decomposed *Thuja* logs in western Canada have been dated to have died at least 1200 years before present [53] implying a decomposition rate-constant below 0.0025 per year as the potential minimum. This suggests that downed CWD longevity can vary almost 400-fold globally.

### Factors influencing *k*

#### Species controls

A total of 114 species in 59 genera of trees have had some estimate of *k* determined, although this is an underestimate in that several tropical studies have reported values for an aggregate of species. As a reference, there are approximately 60,000 tree species in approximately 4370 genera worldwide [54]. Of the approximately 247 plant families containing trees, 29 have been sampled, with the majority (53%) of observations in one: Pinaceae. Of the 36 studies in which multiple species were sampled, all found species differences in *k* which ranged from as little as 30% difference (marginally significant given the relative uncertainty reported) to a 76-fold difference among species. In the Pacific Northwest of USA, a ≈ 10-fold difference has been observed with *Alnus* and *Thuja* having *k*’s of 0.08 and 0.007 per year, respectively [55]. In North Carolina, hardwood species had *k*’s ranging from 0.015 to 0.261 per year, equating to a 17-fold difference [56]. In dry tropical forests a 76-fold difference was observed [57]. Although the species were not identified, [58] reported a 44-fold difference among species. These large differences were typically at sites having genera with high decay resistance present (e.g., *Manilkara*, *Thuja*, *Juniperus*, *Robinia*). However, even when the decay resistance of the genera was low (e.g., *Betula* and *Pinus*), at least a two-fold difference in decomposition rate-constants commonly occurred.

**Fig. 3:**
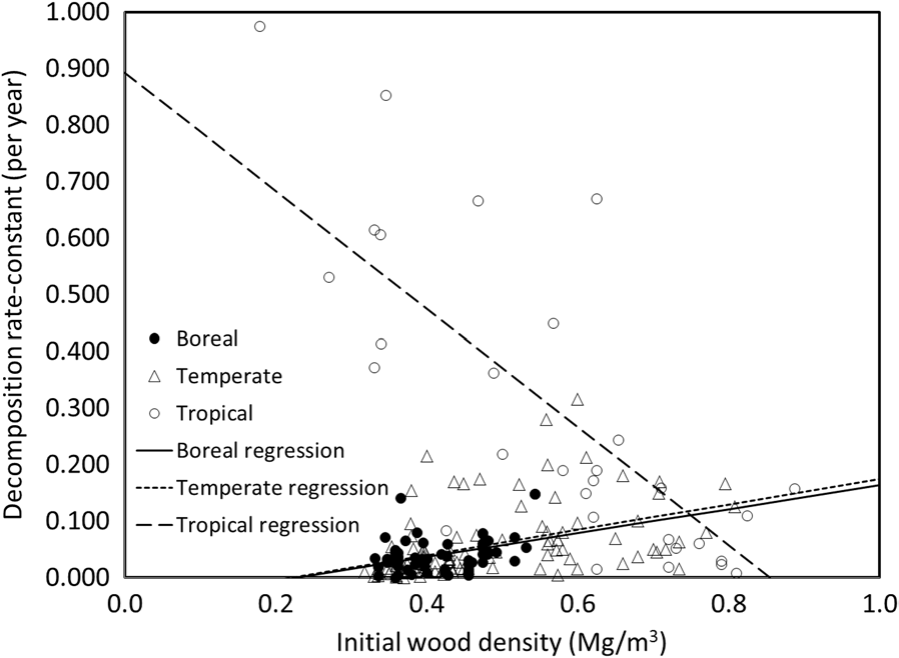
Relationship between initial wood density and coarse woody detritus decomposition rate-constant for boreal, temperate, and tropical species

There are likely multiple factors influencing tree species differences including variations in maximum size, anatomical structure, chemical nature, and the decomposers present. One hypothesis to explain tree species differences is that initial density is related to decomposition rates, with lower density species decomposing faster than denser ones [59]. Regression analysis indicated this to be the case for tropical species; however, the opposite was true for boreal and temperate species (Fig. 3, Table 1). Considering all species together there was no significant relationship between wood density and *k*. The negative relationship in tropical tree species may have had several causes. First, density is often increased by the presence of extractives some of which are toxic to decomposers [36]. Second, softer, lower density wood is probably more attractive to termites and other invertebrate decomposers [60, 61] and the presence of these insects can greatly increase decomposition rates ([31, 37]. The positive relationship suggested by the analysis of temperate and boreal species may have been related in part to the generally lower density of conifer species, which tend to have lower decomposition rates than angiosperms. For example, while some low density temperate woods had relatively high *k*’s, one of the lowest density conifers (*Thuja*) also had one of the lowest values of *k*.

**Table 1:** Linear regression models for the relationship between initial wood density (IWD) and decomposition rate-constant (k)

Biome	A	b	r^2^	P	N
Overall	0.083 (0.037)	0.009 (0.075)	0.001	0.903	199
Boreal	− 0.0480 (0.027)	0.211 (0.065)	0.147	0.002	62
Temperate	− 0.050 (0.023)	0.224 (0.046)	0.187	0.058	102
Tropical	0.892 (0.098)	− 1.043 (0.157)	0.588	< 0.0001	33

The form of the regression model was: *k* = a + b* IWD

**Fig. 4:**
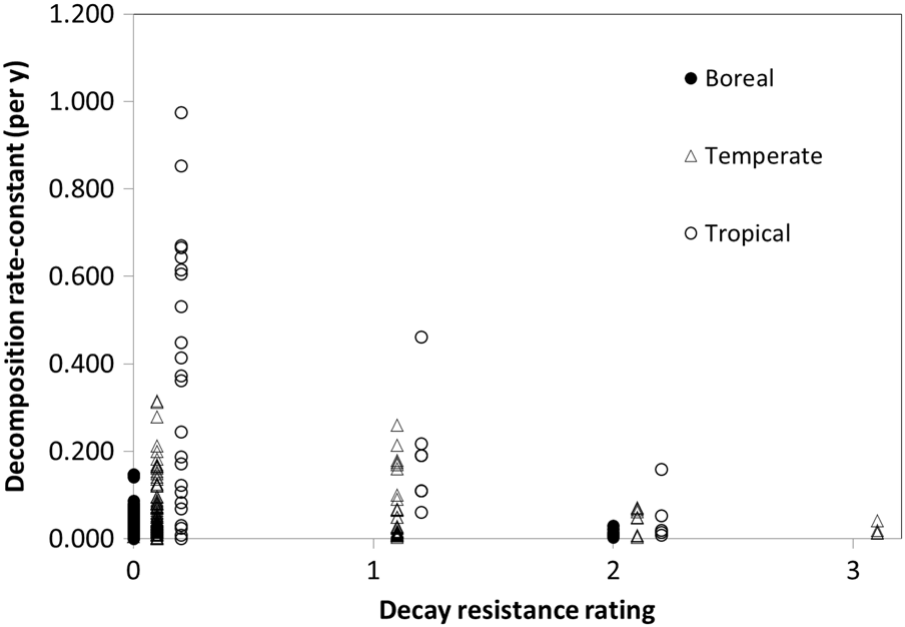
Relationship between decay resistance rating of heartwood (according [65]) and decomposition rate-constant for three climatic zones. Decay resistance ratings: 0 = no to little resistance, 1 = moderate resistance, 2 = resistant, and 3 = highly resistant

While the studies we examined generally did not determine the effects of decomposers (see [62] for an exception and [37] for a general review on invertebrate decomposers), the most obvious cause of interspecific variation in terms of decomposition is related to the presence of decay inhibiting extractives in heartwood. The sapwood for most species at a site decomposes at relatively similar rates, whereas heartwood decomposition rate varies to a much larger degree [63]. Since sapwood and heartwood have very similar anatomical structure and mostly differ in terms of extractives, this difference in tissue type is likely due to the latter. For example, when studying the decomposition of 13 species in Europe, [36] found the strongest negative correlations with decomposition rates for heartwood presence and organic extractives. Although the decomposition of relatively few tree species have been estimated (see above), considerable knowledge exists on the decay resistance of many commercial species used in wood products and construction [64]. We compared the natural durability ratings of heartwood compiled by [65] to the decomposition rate-constants observed (Fig. 4). This indicates there was considerable variation for species with low to no decay resistance and less for those that were highly decay resistant for each of the climatic zones. This may be caused by the fact that as decay resistance increases, it overtakes other factors such as position and climate as controls. However, some of this variation may also be caused by the fact that decay rating systems tend to combine species that are quite different in the least resistant class (e.g., species in the genera *Betula*, *Fagus*, *Populus*, and *Pinus* are all considered the same). Hence, while wood of these genera is not particularly decay resistant as building materials, it did differ in terms of *k*’s often by a factor of 2 or more. Another problem is that decay ratings are based on heartwood, but the proportion of resistant heartwood relative to total wood in a species is also important. For example, although *Quercus* species have similar heartwood decay resistance, *Quercus prinus* boles decomposed almost twice as fast as *Quercus coccinea*, because the former has substantially more sapwood than the latter [66].

#### Effect of piece size

Of the 27 studies that have tested for a size effect (typically expressed as diameter), 22 (81%) identified an effect. The general theoretical prediction is that decomposition should decrease with increasing diameter given that the decrease in surface area to volume ratio with increasing diameter limits exchange of gasses, decomposer access, etc. In addition, the larger the diameter, the more time required to colonize a CWD piece and the more heartwood that is present, both of which could decrease decomposition rates [27]. For 16 (62%) of the studies, *k* decreased with increasing diameter. However, *k* did not always decrease with increasing diameter; 5 (19%) studies found a positive effect of increasing diameter. These were all for open areas following harvest. One found a size effect, but an increase for one species and a decrease for the other [67]. The lack of size effect might be explained by the limited range of diameters examined. The surface area to volume ratio changes most for diameters that are less than 10 cm. For example, between a diameter of 1 and 10 cm the surface area to volume ratio changes an order of magnitude from 4 to 0.4. A similar order of magnitude change occurs in going from 10 to 100 cm (0.4 to 0.04). This indicates that if the CWD diameter range for some studies were too limited (e.g., 10 to 20 cm) or too high (e.g., 50 to 100 cm), a size effect would have been hard to detect. Despite the general theory, there is no reason that decreasing the surface area to volume ratio should always decrease decomposition rates. The positive effect of diameter in dry environments may also have been related to the surface area:volume ratio, but in this environment decreasing surface area to volume may slow drying. Hence, smaller pieces dried faster than larger ones and their decomposition was more limited by insufficient moisture in dry environments.

#### Standing versus downed position

Mortality causes have very different profiles in terms of the degree they create standing versus downed CWD (i.e., the position). Given that standing CWD is more exposed to drying and subject to lower precipitation input, standing CWD is generally drier than downed dead wood. However, given that moisture may be too low or too high to support decomposition, the effect of position could be dependent on the overall climate [21]. Therefore, understanding the degree to which CWD position influences decomposition across a wide range of climates is critical to understanding CWD dynamics across a host of disturbance agents. Unfortunately there have been only 7 studies in which the decomposition rates of these two positions have been compared and reported. Of the 23 species comparisons we found, 7 indicated standing CWD decomposed faster than downed CWD; 3 indicated < 10% difference between positions; and 13 indicated standing CWD decomposed slower than downed CWD. The magnitude of the position effect can be substantial and approached that of the effect of species. Standing CWD decomposition rate-constants can be more than 3 times that of downed CWD (1 case) or less than 1/3rd (7 cases). Lower decomposition rates of standing relative to downed CWD was generally assumed to be related to lower moisture content of the former position. In dry climates this restricts the colonization and activity of decomposers. In some cases standing CWD density and/or mass appeared to slightly increase over time (i.e., the *k* was negative), but this was likely due to shrinkage of the wood and not an actual gain in mass. However, there are also particular genera (e.g., *Betula*) which have bark that appear to retain moisture regardless of climate or position and these species had either little difference between positions or had faster standing than downed CWD decomposition. Alternatively it may be that species with particularly fast colonization by decomposers (e.g., those in the genus *Abies*) were less influenced by the drier condition of standing dead stems than ones with slower colonization.

#### Effect of canopy openness

As indicated under the analysis of size effects, the amount of tree canopy remaining after disturbance can influence decomposition responses. A more open condition will lead to more solar radiation hitting the surface of the CWD leading to potential photodegradation [68] and in addition should lead to warmer and drier conditions that could either favor or retard decomposition. Despite these important potential effects, there were only two studies that specifically examined the effect of canopy cover on CWD decomposition by comparing open versus closed sites. In one case, representing a moist temperate forest, there was little difference between sites that had been clear-cut with those that remained intact [69]. In the second study, representing boreal forests, open sites had higher decomposition rates than closed forest [70]. These differential responses may be due to several factors. For CWD that takes centuries to decompose, open conditions may only be present a small proportion of the decomposition processing time. Moreover, large understory plants can provide substantial reductions in the solar radiation hitting downed CWD despite the lack of tree cover. Hence, closed canopy conditions may be effectively created by plants other than trees. The effect of canopy openness may interact with climate: in cold, wet climates opening the canopy might warm the CWD more than it dries it, thus speeding decomposition [70]. In a dry, warm climate, opening the canopy might dry and heat the CWD to the point decomposition is reduced (e.g., [71]).

#### Climatic controls

**Fig. 5:**
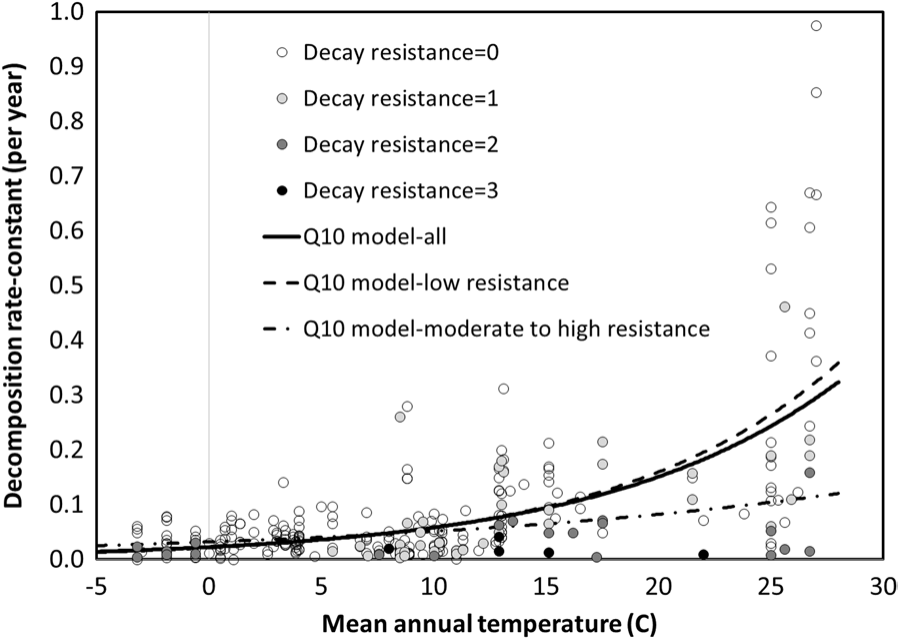
Relationship between mean annual temperature and decomposition rate-constant *k* for species of different decay resistance (0 = lowest; 3 = highest)

Although climate is an important control of CWD decomposition rates [72], it has been difficult to estimate the effect in many broad-scale studies (e.g., [70, 73]. This is partly due to the fact tree species often have limited climatic ranges which suggests that climatic responses within a species might be limited. Examining multiple species over a larger climate gradient avoids this problem, but may confound species effects with climatic ones. There appears to have been a positive relationship between MAT and CWD *k* (Fig. 5); however, there was considerable range of *k* for a given temperature related primarily to species and positional differences. This range appeared to broaden as MAT increases, which suggests that species without decay resistance were more responsive than those with high decay resistance. When all species and positions were examined, non-linear regression indicated a Q10 of 2.50–2.60 with a base value of *k* of 0.058–0.061 per year at 10 ℃ depending on the climatic dataset used (Table 2). After reducing the data to only CWD in the downed position, non-linear regression estimated a Q10 of 2.50–2.59 with a base value of *k* of 0.059 per year at 10 ℃. Further reducing the data to only species with minimal decay resistance gave a Q10 of 2.68–2.75 with a base value for *k* of 0.064–0.065 per year at 10 ℃ depending on the climatic database used. The value of the parameter estimates did not change greatly as the data were narrowed from all observations to just species with low decay resistance; however, the amount of variation explained by the models increased. The fact that the parameter estimates did not vary is likely due to the highly unbalanced sample “design” dominated by downed CWD with low decay resistance. This indicates that an overall analysis of controlling factors could be highly misleading and likely to find that the factors with greater sampling would account for the majority of variance. For example, using the limited number of cases (24) in which standing CWD *k* was estimated indicated a Q10 of less than 1, an indication that as MAT increased standing CWD became drier. The Q10 for species with some decay resistance (64 cases) was 1.57–1.61 with a base value of *k* of 0.051–0.053 per year at 10 ℃. While this is consistent with the hypothesis proposed by [73] that species with a higher decay resistance have a lower Q10, the low sample size leads to considerable parameter uncertainty. The few species examined with very high decay resistance suggest that the Q10 approached 1; however, without more data, particularly in warmer climates, this response was difficult to estimate with any certainty. Additional data, particularly in the tropics, would give considerable insight into the generality of Meentemeyer’s [74] hypothesis of the substrate quality-climatic interaction being the key control of global decomposition patterns.

**Table 2:** Q10 relationship between mean annual temperature (MAT) and decomposition rate-constant (*k*)

Model	k10	Q10	r^2^	p	N
All/reported^a^	0.058 (0.006)	2.60 (0.21)	0.557	< 0.001	289
All/WC2^a^	0.061 (0.006)	2.50 (0.20)	0.542	< 0.001	289
Downed/reported	0.059 (0.007)	2.59 (0.22)	0.562	< 0.001	263
Downed/WC2	0.062 (0.007)	2.48 (0.21)	0.546	< 0.001	263
Standing/reported	0.013 (0.008)	0.575 (0.33)	0.500	< 0.010	24
Standing/WC2	0.013 (0.009)	0.509 (0.40)	0.500	< 0.010	24
Low decay resistance/reported	0.063 (0.007)	2.75 (0.23)	0.620	< 0.001	228
Low decay resistance/WC2	0.065 (0.007)	2.68 (0.22)	0.601	< 0.001	228
Some decay resistance/reported	0.051 (0.008)	1.61 (0.25)	0.552	< 0.001	64
Some decay resistance/WC	0.053 (0.008)	1.57 (0.24)	0.548	< 0.001	64

The general form of the model was *k* = k_10_Q10^((MAT−10)/10)^

^a^Reported indicates that the MAT reported by a study was used; WC2 signifies WorldClim 2 data

**Fig. 6:**
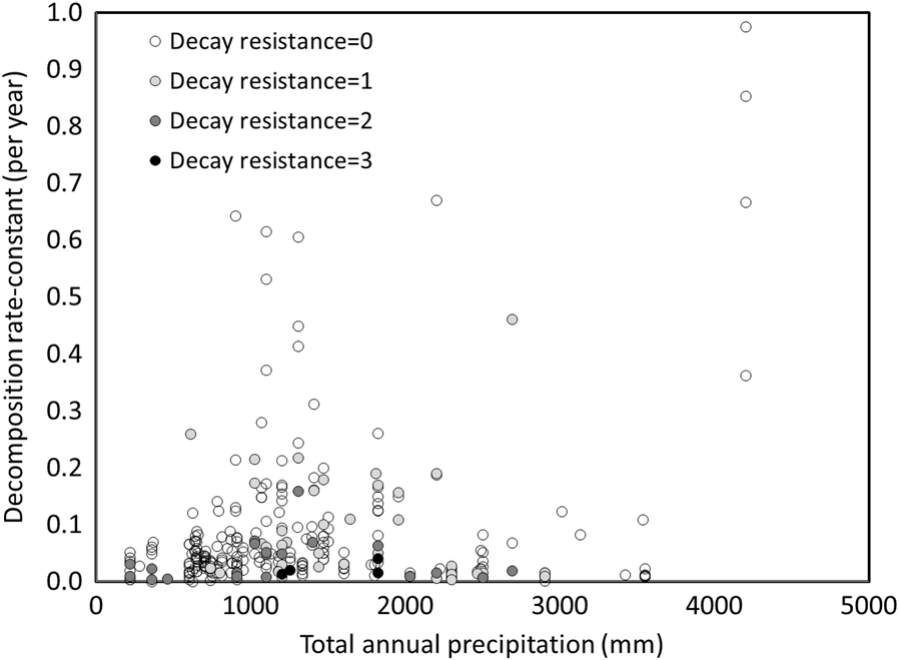
Relationship between mean total annual precipitation and decomposition rate-constants of coarse woody detritus for species of different decay resistance (0 = lowest; 3 = highest)

Regression analysis indicated there was no significant linear or polynomial relationship between *k* and TAP for any of the climatic databases we examined. The range in *k* appeared to increase greatly once precipitation exceeds 1000 mm (Fig. 6). However, this could also be related to the diversity of species found at these wetter sites as well as the general correlation between MAT and TAP for the sites of CWD decomposition studies (Fig. 2). From this analysis we do not necessarily conclude that precipitation per se is an unimportant control of decomposition; rather that other aspects such as seasonality and moisture balance should be explored more fully.

#### Interaction of factors

Given the unbalanced nature of the overall experimental design of the data we compiled, we largely examined, with several notable exceptions such as the effects of initial wood density and climate variables, the relative range of *k*’s for a given factor. In comparing factors one can consider either the degree it influences the CWD decomposition process or the uncertainty in the range within a factor. Based on our analysis it would appear that the relative range within a factor, in terms of influence, ranks approximately as follows: species > climate > position > size > canopy openness. We acknowledge that for canopy openness, the paucity of data likely underestimate the potential range in this factor. We rank the factors examined in terms of uncertainty as follows: canopy openness > position > species, climate, and size. Our ranking of uncertainty is strongly influenced by how widely a factor has been examined. The lower relative uncertainty related to species, climate, and size should not be interpreted that additional information, particularly at the mechanistic level, is not warranted. Rather, it means that so little data on canopy openness and position exist that insights into sign and magnitude of their controls are scant.

While assigning the individual factors importance in terms of the degree of process control and uncertainty has value, it misses a major finding of our analysis: the evidence that many, if not all the factors interact in important ways. Hence, while species rates of CWD decomposition in tropical forests can range up to 76-fold, MAT can restrict this range to several-fold in boreal forests. Similarly, if the relationship we found between MAT and standing CWD decomposition rates were proven real, then warming and drying of the climate would cause standing CWD to decompose more slowly than downed CWD to the degree that the two positions would markedly diverge. Position is also likely to interact with size, particularly in drier climates, with small diameter standing CWD being more limited by low moisture than larger diameter standing CWD. Although the effect of canopy openness is not clear, it is likely that climate, size, and position interact with this factor. This suggests that responses as divergent as a decrease, an increase, and no response of decomposition rate to increased canopy openness are all possible. Our conclusion is that while additional information about individual factors is warranted, the major uncertainty is associated with interactions.

### Time course of disturbance-related fluxes

To give a sense of the potential variation in the time course of carbon release following forest disturbance, we used decomposition data to model two cases: a hurricane in which the majority of CWD created was downed, and a beetle-kill in which the majority was created as standing CWD. In both cases, we assumed that all decomposition losses were associated with respiration and not fragmentation and leaching. We acknowledge that both of the latter occur, but for illustrative purposes we assumed respiration was the only pathway of loss.

#### Carbon release after hurricane

**Fig. 7:**
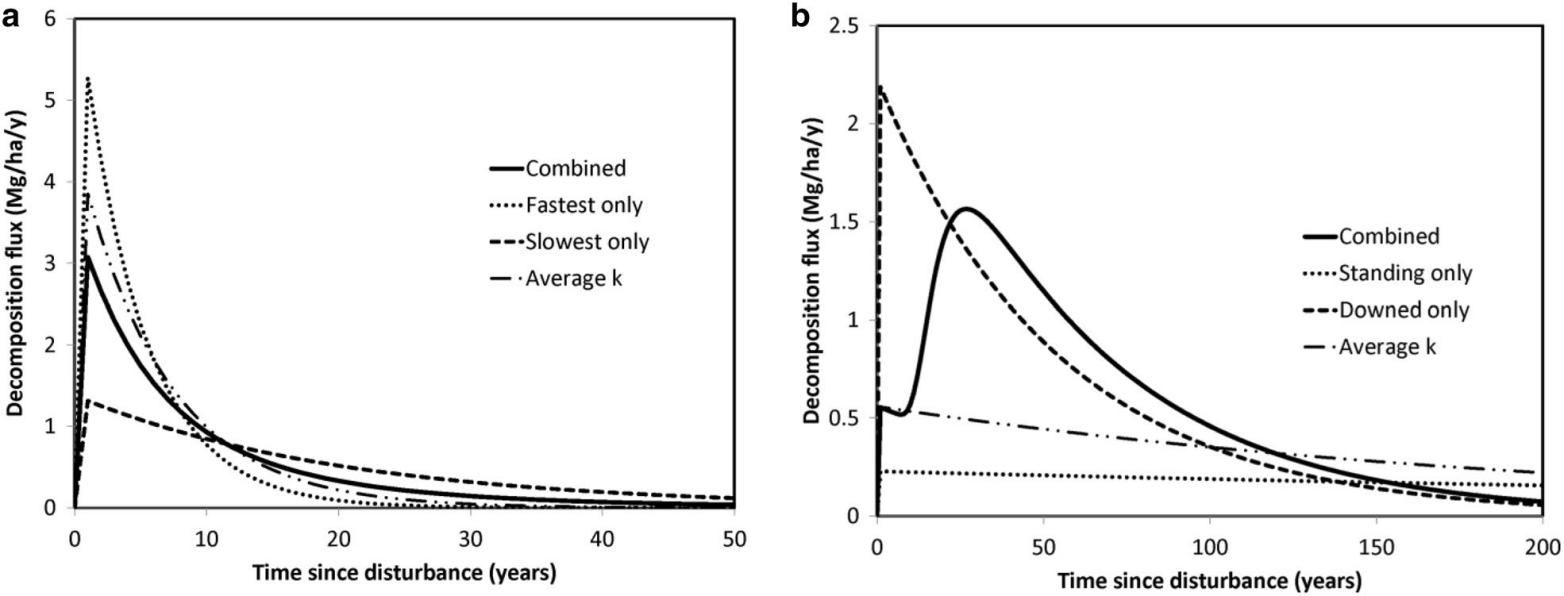
Temporal patterns of decomposition-related carbon flux from CWD following major disturbance: examples of **a**. Hurricane in North Carolina and **b**. Beetle-kill in Colorado. The combined line indicates the flux associated with the mix of species or positions. The average *k* decomposition flux line is based on the initial weighted average rate-constant and assumes the proportions do not change over time. (See text for details)

Hurricane Fran, which occurred in 1996, added 48 Mg of dead tree mass per ha [75]. We used the composition of the forest in North Carolina found by [75] to estimate the proportion of species killed and then applied their *k*’s to estimate the respiration flux over time. This indicated that many genera decomposed within the 20 year measurement period (*Acer*, *Carya*, *Fagus*, *Liriodendron*, and *Liquidambar*), but others (*Juniperus* and *Quercus*) would take at least twice as long. The effect of the species mix changed after disturbance causing the decomposition rate-constant to “evolve” in a non-linear manner over time. Initially, the weighted average *k* for this site was 0.151 per year, but at 9 years after disturbance it was 0.09 per year and at 50 years it was 0.057 per year. This highlights the problem of using the average *k* of species: the overall *k* converged over time toward that of the slowest decomposing species. Similarly the decomposition flux evolved over time: for 9 years after the disturbance decomposition-related flux was larger than if the forest were entirely comprised of slower decomposing genera, but after that point in time the flux was higher than if the forest were entirely comprised of faster decomposing genera (Fig. 7a). Using the initial average *k*, even if weighted for species abundance, did not address these non-linear effects. The large range in species-related decomposition observed suggests that for many locations the mixture of the species dying can have a very large impact on subsequent release of carbon. Specifically, when abundant species differ by an order of magnitude in terms of *k*’s (which based on our analysis was not an uncommon situation), the potential for non-linear temporal patterns of carbon release was high. Although we did not examine the effect of size on temporal dynamics, we expect the effect on evolution of the *k* would have been similar to that of species.

#### Carbon release after beetle-kill

Our beetle-kill example occurred in Fraser Experimental Forest, Colorado where between 2004 and 2009 ≈ 100 Mg/ha of live tree biomass was killed, a live store typical of an older stand in these forests [76, 77]. Since there was some downed CWD before the beetle-kill, we assumed an initial downed store of 20 Mg/ha. We also assumed that standing *Pinus* CWD began to fall after 10 years (consistent with our observations during sampling) and that it would take an additional 30 years for the standing CWD to fall completely [78]. Based on our unpublished *k* estimates [52] (Additional file 2: Table S-1), the decomposition rate of standing dead *Pinus* trees was 10% of the downed ones. This difference in positions was likely caused by the dry climate present at the site, which experiences an extended summer drought. Because of this difference in standing and downed *k*’s and the lag in snag fall, a delay in the decomposition-related flux occurred, with the peak loss to the atmosphere 25–28 years after the disturbance (Fig. 7b). In this example the decomposition-related flux was highly non-linear and did not stay within the bounds defined by the loss that would have occurred if all the CWD remain standing or start in the downed position. As with the North Carolina hurricane example, the *k* evolved over time as the mixture of standing versus downed CWD pools changed. Therefore, using an average *k* gives a very misleading prediction of decomposition-related fluxes both in terms of value, but also in terms of temporal complexity. Although in this example decomposition rates increased with snag fall, there are likely situations in which the opposite is true. On hydric sites dead trees may become waterlogged after they fall slowing or arresting decomposition. For example, in moist boreal forests with relatively fast moss growth, downed wood can be buried and accumulate over time [47]. If standing CWD *k*’s are also low, then it is possible that decomposition-related fluxes would be minimal after disturbance at these sorts of sites. It is also possible for disturbance to alter hydrology sufficiently to create site conditions that favor waterlogging of CWD [79].

### Implications of results for modeling

The previous examples indicated that the differences in species and positions can cause the ecosystem level *k* to evolve over time. In this section we further examine the implications of this behavior on modeling the trajectory of carbon release from CWD from a wider set of cases. It should be noted that we did not examine the effect of size differences; however, these would be analogous to that of species although of smaller magnitude given that difference in sizes was generally less than an order of magnitude.

#### Species influences

**Fig. 8:**
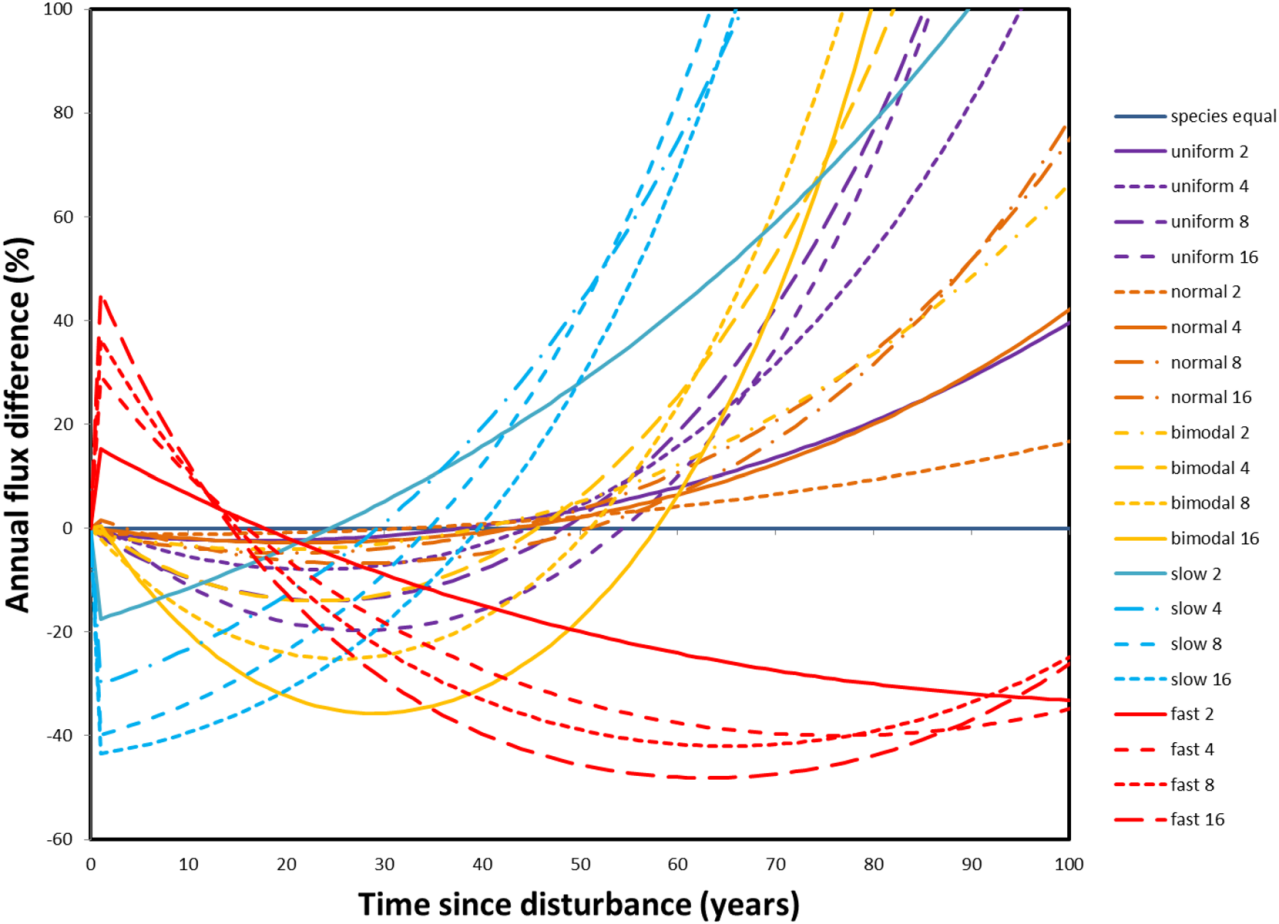
The relative annual carbon flux difference from the case in which all species have the same *k* value. In the legend the first word indicates the species abundance distribution used and the number indicates the ratio of *k*’s for the fastest and slowest species

Substantial relative differences occurred from the reference case in which species all had the same *k* (Fig. 8). As might be expected, when the slowest decomposing species was most abundant the initial flux was between 18 and 45% slower than the reference case depending on the fastest to slowest *k* ratio. Conversely, fluxes after 20-40 y were in some cases over 100% higher. The opposite temporal pattern occurred for distributions in which the fastest species was most abundant. While the effects of the other distributions were less dramatic, there were also notable deviations from the reference case even when distributions resulted in an average *k* close to that of the reference case (e.g., uniform, normal, and bimodal distributions). Although the absolute differences between the cases after 50 years was low because ≈ 8% of the carbon remained at that point in time, the deviations occurring earlier were large in both a relative and absolute sense (Additional file 1: Figure S6). This indicates that one needs to model dead wood carbon release in a manner that accounts for both the differences in *k* among species (or classes of species) and the relative abundance of the species.

#### Position influences

**Fig. 9:**
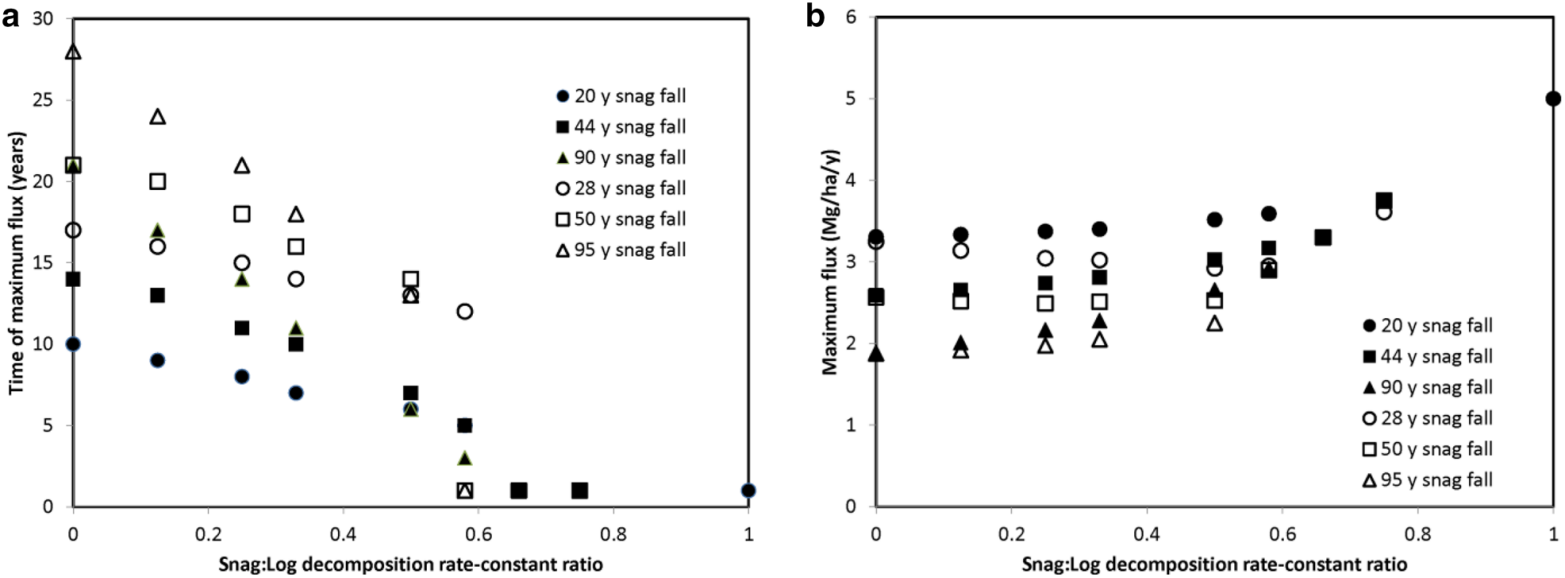
Effect of snag:log decomposition rate-constant ratio and timing of complete snag fall on the time (**a**) and value (**b**) of the maximum flux from mortality caused by a disturbance in the case in which snag *k* is less than that of logs

**Fig. 10:**
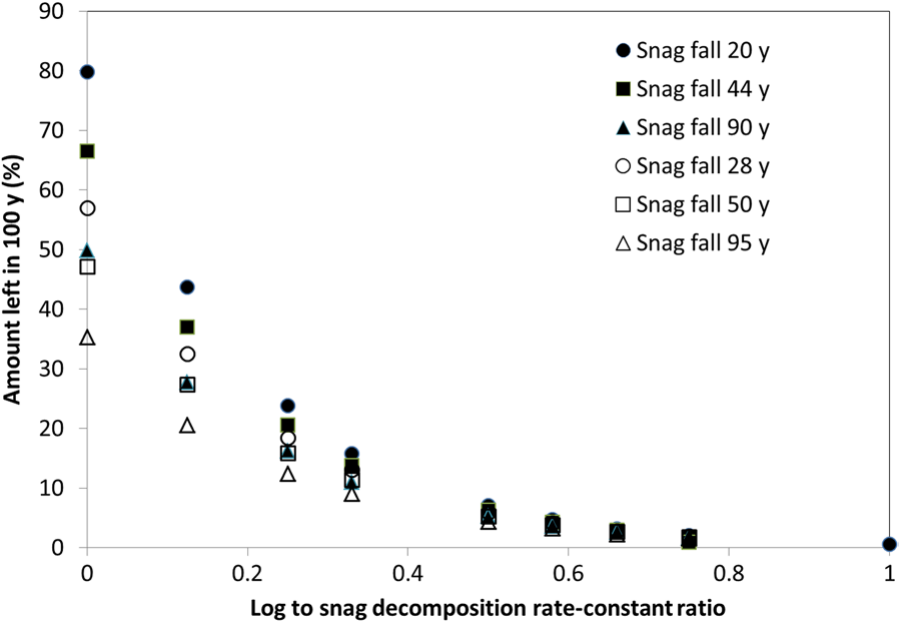
Effect of log:snag decomposition rate-constant ratio and timing of complete snag fall on the amount of carbon in CWD caused by a disturbance left after 100 years in the case in which snags *k* is higher than that of logs

When snag *k* was lower than that of logs, the time to reach the maximum carbon flux exhibited a lag that increased as the difference between the two positions in terms of *k* increased and as the time for snags to completely fall increased (Fig. 9a). The occurrence of a lag before snags begin to fall also increased the lag in timing of the maximum flux even when the time of complete snag fall was similar. The value of the maximum carbon flux was also influenced by the rate of snag fall, but not the snag to log *k* ratio, at least for values less than 0.5 (Fig. 9b). When the snag *k* was higher than that of logs a very different response was observed because there was no lag in the maximum flux (Additional file 1: Figure S10). The amount left after 100 years (i.e., the time when 99% of the carbon would have been released with a *k* of 0.05 per year) indicated that substantial amounts of carbon can remain in this set of cases (Fig. 10). For example, in the case in which log *k* approached 0 per year, between 35 and 80% of the carbon was not be released within 100 years with the amount unreleased decreasing as the snag fall rate decreased. This analysis indicates that to correctly estimate the magnitude and timing of the flux correctly one must segregate by CWD position if the *k* of snags and logs differs more than a factor of two and snag fall takes a decade or more. It should be noted that while this analysis focused on standing versus downed CWD, similar responses would likely have occur if one contrasted downed and buried CWD.

### Effect on carbon balance

**Fig. 11:**
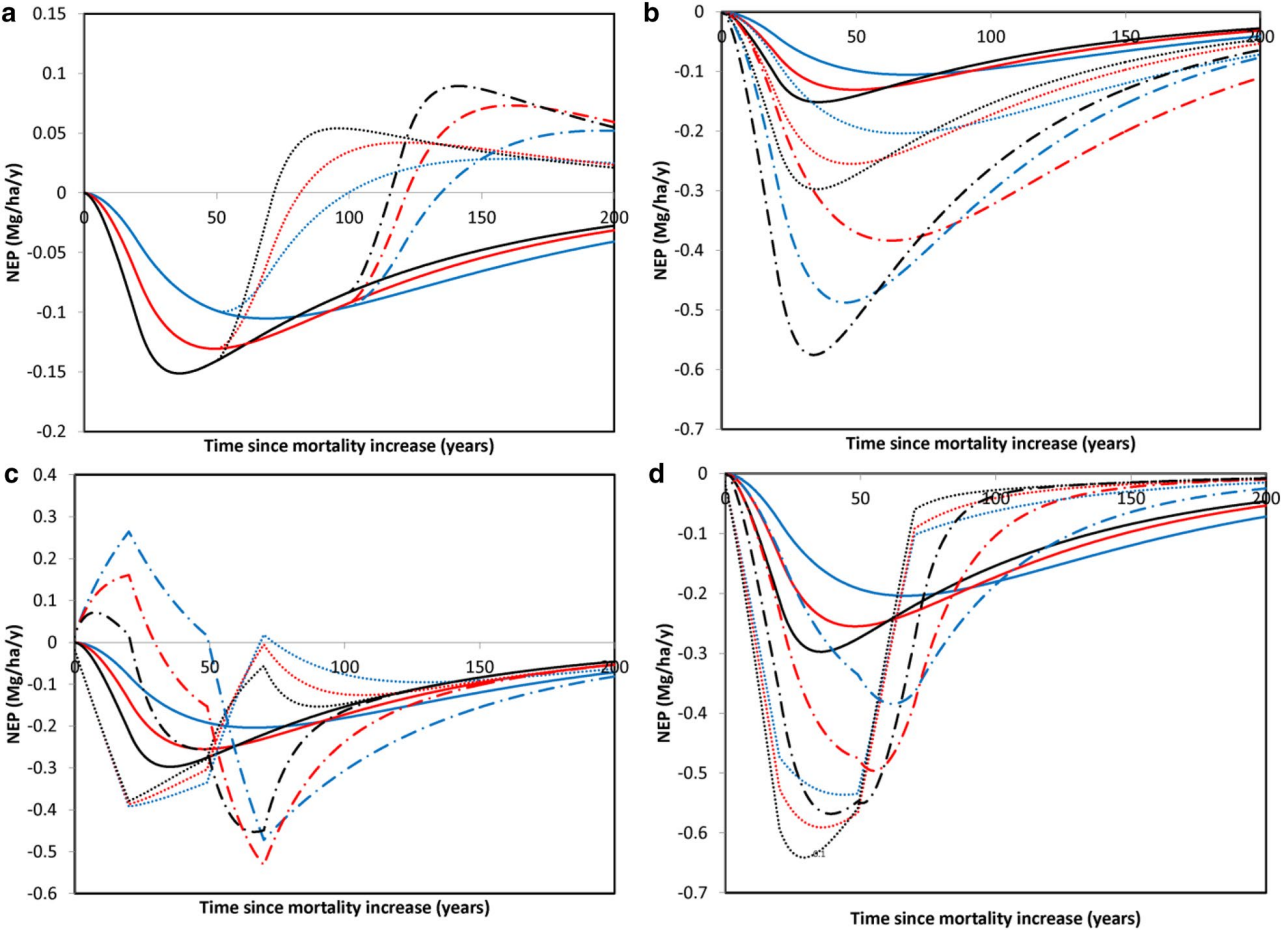
Hypothetical responses of net ecosystem production (NEP) to increases in mortality as mediated by CWD decomposition. For each line the *k* is blue − 0.025 per year; red − 0.05 per year; black − 0.10 per year. **a** Duration of 10% mortality increase: dotted 50 years, dot-dashed 100 years, solid line > 200 years. **b** Magnitude of permanent mortality increase: solid 10% increase, dotted 20% increase, dot-dashed 40% increase. **c** Change in decomposition rate for 20% increase in mortality: dotted 20% increase for 50 years, dot-dashed 20% decrease for 50 years, solid no change. **d** Changing NPP and decomposition for 20% mortality: dotted 20% decrease in NPP for 50 years, dot-dashed 20% decrease in NPP and decomposition rate-constant for 50 years, solid no change in *k* or NPP

When we varied the duration of the mortality increase (in this particular experiment the increase was set to be 10% higher than the base level), we found that if the mortality increase lasts the entire simulation period (200 years), then NEP remains negative during the entire period (Fig. 11a). In contrast, when elevated mortality was a temporary phenomenon (50 or 100 years), then the period of negative NEP was followed by a positive period as the forest landscape recovered. Regardless of the duration of elevated mortality, the timing was strongly influenced by the decomposition rate-constant: forests with higher decomposition rate-constants responded faster than those with lower ones. As might be expected, the magnitude of the mortality increase influenced the magnitude of the NEP response; however, the decomposition rate-constant controlled the timing of the response (Fig. 11b). Specifically, the most negative value of NEP was reached first by the forest with the fastest decomposition. It also approached zero (i.e., carbon balance) earliest, although in no case was a NEP of zero actually reached, suggesting that the impacts of increased tree mortality could last centuries in many boreal and temperate forests. The response of decomposition to mortality had a major impact on temporal changes in NEP (Fig. 11c). If, on one hand, the decomposition rate-constant decreased because, for example, standing dead CWD was created or open conditions reduced decomposition rates (in this case by 20%), then the initial response to increased mortality would be a positive increase in NEP at least until conditions favoring decomposition return. Once decomposition resumed its initial rate-constant value, NEP would become more negative than when decomposition rate-constants remained constant. If, on the other hand the decomposition rate-constant increased by a similar amount as mortality (20%), the NEP response would be more complex. Specifically, at first it was more negative than when decomposition rate-constants remained constant. This was followed by a period in which it was less negative, due to the fact that CWD was re-accumulating following the period of elevated decomposition. Changes in decomposition could interact with changes in NPP as well (Fig. 11d). If NPP also decreased temporarily (in this case by 20%) due to increased mortality (perhaps due to lags in tree regeneration), then NEP became much more negative than when NPP remained constant. Interestingly, if decomposition rate-constants also decreased over the same period, then these net carbon losses were somewhat mitigated as decomposition-related losses reduced.

Our model of carbon balance, while lacking detailed mechanisms, does include the range of changes in mortality, NPP, and decomposition that can be anticipated. Our overall conclusion from these preliminary modeling experiments is that while the magnitude and duration of elevated mortality will be important in controlling future forest carbon balances, the response of CWD decomposition to increased mortality will be equally critical as it is a major determinant of the carbon balance (i.e., magnitude and timing of net carbon loss) of forested ecosystems. In particular, if disturbance influences the rate-constant of decomposition (i.e., *k*) then the response of carbon balance would be quite complex.

### Modeling approaches

Our intention was not to conduct a detailed, exhaustive review and evaluation of how models consider the process of CWD decomposition; therefore below we examine a range of examples using general aspects of their structure to assess the degree to which they would potentially mimic the CWD carbon dynamics and balance as identified via our study of CWD decomposition. These examples were selected from models that are currently used in high-level scientific exercises and/or carbon accounting, assuming that these examples represent the *status quo* of carbon modelling in the respective areas.

#### How models have been used

Under the Kyoto Protocol, forest models have been used to estimate the Forest Management Reference Levels to be used in the second commitment period. Under current EU legislation a “Forest Reference Level” is to be used in the EU member states for accounting purposes in the period 2021–2030. Models used by the European Commission and a large number of member states include, among others, EFISCEN (European Forest Information Scenario model), GLOBIOM (Global Biosphere Management Model), G4M (Global Forest Model), CBM-CFS3 (Carbon Budget Model of the Canadian Forest Service), EFDM (European Forestry Dynamics Model), FVS (Forest Vegetation Simulator), and YASSO (please see [80] and [81] for details).

#### Review of model examples

The models could be sorted into three groups: (1) forest models usually used to specifically simulate forest development including management, (2) vegetation or land use models (e.g., Dynamic Global Vegetation Models), and (3) soil models, sometimes run as part of a larger framework or as a module within another model (Additional file 1: Table S-2).

According to the documentation available, of the models recommended for use in the EU [81], two (G4M and GLOBIOM) did not specifically include CWD decomposition and two (EFDM and EFISCEN) referred to the soil model YASSO to simulate this process. The latter contains five soil organic components three of which (i.e., extractives, celluloses, and lignin-like compounds) receive inputs from fine and coarse woody litters. This method is used to model the course of both woody and non-woody litter decomposition [82]. The decomposition rates of the YASSO compartments are adjusted by air temperature and precipitation. However, by aggregating all tree species this model cannot address changes in tree species composition or size structure if they differ from the parameterization at the beginning of the simulation. Moreover, it cannot address changes in the dominance of dead tree positions that different kinds of disturbances could create. In contrast, CBM-CFS3 [83], also recommended in the EU, allows for a more detailed simulation of both standing and downed FWD and CWD that are separated into hardwood and softwood classes, but not by tree species per se; although this could in theory be addressed to some degree by ecosystem-specific adjustment factors. According to the documentation available, FVS [84, 85] separates standing and downed CWD. Its structure offers the most potential to adjust decomposition rates of various dead matter pools because, at least for living pools, it tracks pools by species and by size.

For most of the models used in ISIMIP, no detailed information about the treatment of decomposition could be consistently derived from the documentation provided by the modelers. Where such information could be identified, dead wood was usually treated as a form of litter or directly added to a “labile” soil carbon pool. Decomposition was typically tracked in 2–4 soil carbon pools with different turnover rates and transfer rates between these pools. Exceptions are LPJ-GUESS (“Lund Potsdam Jena General Ecosystem Simulator”) with 11 soil organic material compartments that include CWD and FWD [86] and 4C (“FORESEE—FORest Ecosystems in a Changing Environment”) [87], whose soil module contains five soil depths, including a humus layers that includes all forms of litter including CWD. LPJ-GUESS and other models do not distinguish tree species, but include “plant functional types”. Given the wide variation of *k* within even the same tree genus, this approach may not fully capture CWD decomposition, especially if disturbances affect different trees species to different magnitudes hence change the mixture of species in the dead wood pool. These models also do not include the effects of size or position on CWD decomposition and could not be used to assess the impact of how disturbance influences these factors. Models that used several pools with different transfer rates varied these decomposition rates due to soil moisture, temperature, and, in three models, nitrogen availability, or pH (4C). Thus, they included some of the influences identified in our analysis, but their approach of using a single cascade of pools aggregated by species, size, and position would be very susceptible to weighing bias and errors, if and when the composition of dead wood input changed over time. We are aware that incorporating all factors presented in our analysis in decomposition modelling at a global level is not practicable (especially species-related effects or microclimatic factors like stand openness), but we caution that changes in tree species composition, tree size, and the position of dead trees will have influences on the future carbon release from dead wood pools and should be considered in future analyses and interpretations of modelling results.

### Future directions of dead wood decomposition research

#### Entering a new phase of research

Remarkable progress has been made in the last few decades in estimating the decomposition rates of CWD and identifying some of the major controlling factors. The first estimates in our compiled database dates from 1970 with the majority appearing after 1990. This early phase of CWD decomposition science might be described as largely opportunistic and observational. That is, these studies largely took advantage of existing situations to make preliminary estimates of this process, often with sample sizes < 10 (we acknowledge there are some notable exceptions). Although we expect that additional estimates of this nature will continue to accumulate in the future, we believe that sufficient information exists to envision a new phase of this science which could be more systematic, experimental, and replicated.

#### Methodological approaches

As noted above, the majority of estimates have come from chronosequences. This makes perfect sense in that the decomposition of individual CWD pieces can take many human lifetimes. We expect that this approach will be used in the future; however, it does have major limitations that need to be considered. First, the initial condition (i.e., position, size, density, and presence of previous decay) of the pieces can be difficult to determine, particularly when decomposition is extensive. This makes determination of mass and volume loss very challenging. At best one can approximate initial volume and mass which unfortunately introduces uncertainty into decomposition rate estimates. At worst, use of remaining density without adjusting for volume loss can bias decomposition rates downward [88]. To gain additional insights into CWD decomposition dynamics, it would be helpful if changes in volume, density, and mass were examined and reported. Some of the difficulty in determining the effect of size in chronosequences is that sometimes there are high levels of uncertainty regarding the initial size. Second, by substituting space for time the environment can differ not only in space, but also in time: that is the environment in the near past might be different than the far past even in the same place. The third is that one has to sample the pieces that exist and this makes controlling factors and experimental designs difficult. Many of these problems can be overcome by the use of time series. However, this brings one back to the challenge of conducting experiments and observations over multiple human lifetimes. We believe that in limited cases it worth the effort to create high resolution time series studies (see [35, 89]); however, it is hard to envision this as the main approach employed in the future. Perhaps the best practical compromise would be to use the decomposition vector method in which a range of decay stages are observed over a relatively short period of time (i.e., 5–10 years). This approach shortens the time required for study, examines actual temporal changes, and allows one to examine whether decomposition rates themselves are a function of decay state. The latter would be extremely helpful in coupling to large scale inventories of CWD, which usually identify the decay class/stage. This coupling would allow one to estimate CWD decomposition losses for very broad regions, something that decomposition studies of individual pieces are challenged to do because they depend on knowledge of species and decay state mixtures and not just the differences among these groupings. Broad scale inventories could also assist decomposition science. Given the small sample sizes involved in decomposition estimates, robust estimates of the residence time in decay classes are rarely provided in decomposition studies. Broad scale inventories, particularly using revisited pieces [43], could provide the large numbers required to provide robust estimates of decay class residence times that would help decomposition vector studies (e.g., [44, 90]). Broad scale inventories can also provide robust estimates of standing dead fall rates [91]. Regardless of how well standing versus downed CWD decomposition rates are quantified, it cannot be fully taken advantage of until the rate of transfer between these two forms of CWD is better quantified.

While we have examined CWD as an aggregated black box using the rate-constant of decomposition (*k*) as the key variable, CWD is not a homogeneous substrate and external losses are not the only processes involved during decomposition. Examination of tissues within CWD and how these are transformed by decomposers would help explain species and size effects as well as non-linear changes in how CWD pieces decompose over time. For example, the degree to which the presence of white- versus brown-rots in CWD [92–94] influences the decomposition process needs to be better explored given that decomposition by brown rot can result in a substantial amount of lignin-rich residual material entering the “soil” pool [95]. Hence, the inability of brown-rots to significantly degrade lignin would form the mechanistic basis of asymptotic decomposition models. Finally, a better understanding of the pathways by which carbon leaves CWD needs to be gained. As [33] point out, it cannot be assumed that all losses in CWD mass are a direct result of respiration. However, that does not mean that fragmented or leached carbon is not respired; understanding the situations in which fragmentation and leaching decreases versus increases respirational losses are critical to fully understanding how increased mortality influences atmospheric carbon.

#### Improving experiments

Experimental manipulation of CWD [e.g., 35, 62, 89] is challenging given its size and expense. However, we believe that experimental designs could be substantially improved in the next phase of this science. The effect of tree species could be better understood if a wider range of species were examined, with more attention paid to examining species with the full range of decay resistance at a site. This would allow one to confirm that decay resistant genera respond less to climate than those with limited decay resistance. As a whole, species in the family Cupressaceae would be a logical starting point for genera with high decay resistance that span a wide range of biomes from boreal (*Thuja*) to subtropical (*Cunninghamia* and *Taxodium*) to woodlands (*Juniperius*). One could also take advantage of genera such as *Robinia* that have been planted widely and would offer a highly decay resistant species for comparison. The effects of size, as indicated by diameter and length, could be better understood if a wider range of sizes and conditions was examined. In this regard, it would make sense to combine studies of fine woody detritus (FWD) with CWD because size effects are most pronounced in smaller sized pieces. There is sufficient evidence to suggest that there is no single size effect, rather it differs with canopy openness, the overall climate, and CWD position. This suggests that far more combinations of conditions have to be examined to unravel this complexity. Fortunately FWD is much more amenable to experimental manipulation and this may make experiments that compare positions or effects of canopy openness more common in the future. Position effect experiments for CWD may be difficult to conduct given the danger and difficulty in creating standing dead trees. In this case it may be more practical to find situations in which standing dead trees have been created (e.g., wildfires or insect outbreaks) and then fell subsets of these trees to create the downed CWD for comparison. In addition, these felled trees can provide estimates of the standing CWD initial condition.

#### Determining climatic effects

Determining the effect of climatic factors on CWD decomposition has been challenging. While it may be possible to manipulate the amount of moisture or temperature, the most expedient approach may be to examine genera that grow under wide temperature and precipitation ranges. Tropical forests remain understudied relative to boreal and temperate ones (Fig. 2). Increasing information for tropical forests will be important for several reasons: (1) they represent the upper limit of temperatures for forests, (2) have a diverse tree taxa that is generally distinct from the ones in boreal and temperate forests, and (3) have higher macroinvertebrate decomposer abundance than their boreal and temperate counterparts. The range of climates that has been examined is quite wide; however, more attention to warmer, drier conditions (e.g., typical of woodlands) and warmer, wetter ones (e.g., subtropical) would reveal more about climatic controls and their interaction with other factors such as position. We found two genera, *Betula* and *Pinus*, that could prove useful and others with wide climatic ranges such as *Populus*, could be targeted. Given the interaction between decay resistance and climate, care needs to be taken to assure that the former is controlled. This is not an issue for genera lacking heartwood decay resistance (e.g., *Abies*, *Betula*, and *Populus*), but can be problematical for those with even moderate decay resistance in heartwood if the proportion of heartwood in stems varies within a genus (e.g., *Pinus*). To circumvent this problem, it may be necessary to separate sapwood and heartwood decomposition for genera of the latter type.

In addition to an improved experimental design regarding climate, more attention needs to be placed on finding more meaningful climatic indices. While MAT is often used, it might not be as insightful as indices that include seasonal effects and periods of peak biological activity such as degree-days. Although total precipitation approximates the effects of moisture, examining the effect of either the seasonality of precipitation or a more direct index of moisture would likely provide more mechanistic insights on climatic controls as well as interactions related to size, position, and canopy openness. It also has to be recognized that moisture is not necessarily a “fixed” site property in that disturbances can make sites effectively drier or wetter depending on the ecosystem.

#### Modeling CWD decomposition

Modeling of CWD decomposition occurs at several levels. Modeling decomposition losses at the level of individual trees has largely been based on the single negative exponential model. If data on for separate tissues or layers were available, a multiple negative exponential model could be employed with the benefit of predicting non-linear trends [27]. Non-linear trends could also be predicted using models with temporal lags [34], but precisely fitting these would depend on data with higher temporal resolution early in the decomposition process than has generally been available. The opposite problem occurs for asymptotic models: greater temporal resolution of the latter stages of decomposition is required to determine the asymptote.

Our analysis of the time course of carbon release indicates that the way CWD decomposition is modeled at higher levels may have to be reconsidered if underlying driving factors and the complexities of the temporal pattern of carbon release and carbon balance is to be captured (see Fig. 11 for examples). First, many global models predict CWD decomposition using lignin, cellulose, and other organic fractions (often only separated in two or three pools of different “decay resistance”); however, very few of the empirical studies we examined explain their results using these factors. Instead, factors such as species, decay resistance as related to extractives, size, position, and microenvironment were used. This implies a fundamental disconnect between models and empirical studies that needs to be addressed. Second, while one may need to aggregate as much as possible when modeling, this has to be done in a manner that allows the overall decomposition rate-constant to evolve over time. Aggregating species with markedly different decomposition rate-constants, which can differ at some sites by at least an order of magnitude, can lead to systematic overestimation of fluxes immediately after disturbance and underestimation later (Fig. 7). Moreover, to capture the response to climate one might also have to consider the effect of species. Our analysis and that of others (e.g., [73]) suggest that species with high decay resistance respond less to changes in MAT than those with lower decay resistance; hence the “average” temperature response at a site depends on the species mixture. In addition, the species mixture can change over time in response to a warming climate and one cannot assume that the decay resistance of the replacement species will be the same as the original ones. Given that it can take decades to centuries for CWD to decompose, these legacy effects could influence carbon dynamics for a considerable time.

In regions in which standing and downed CWD have substantially different (an order of magnitude or more) decomposition rate-constants, using an aggregated average can entirely miss the non-linear nature of the decomposition related flux. While the degree to which these aggregation errors express themselves is likely dependent on the system being studied as well as the scale, it cannot be routinely assumed that decomposition rate-constants and their related fluxes are static proportions over time. Future modeling needs to address these issues, but future decomposition studies also need to provide a stronger basis for aggregated modeling systems by reporting non-aggregated values to the extent possible.

## Conclusions

We examined estimates of CWD decomposition rates collected over the past ≈ 50 years around the world to determine the effects of species, size, position, canopy openness, and climate. Based on the 295 estimates compiled, we found strong evidence that each factor had an influence with their combined effect leading to orders of magnitude differences in the decomposition rate-constant implying a lifespan of CWD as short as 3 years and as long as at least 750 years. The effects of differences related to species and position (and likely size) can lead the aggregated decomposition rate-constant to evolve over time resulting in non-linear temporal patterns of carbon release. This indicates that while considerable progress in understanding the release of carbon via CWD decomposition has been made, a more systematic, experimental, and replicated approach is warranted if the impact of increased tree mortality are to be fully understood and anticipated in regards to C cycling and broader ecosystem functions.

## Supplementary information

**Additional file 1.** Supplemental text, figures, and table.

**Additional file 2.** Spreadsheet containing data used in analysis of decomposition rate-constants.

## Data Availability

The data set of compiled decomposition rate-constants and controlling variables is available in additional al online materials in the form of an Excel spreadsheet (Additional file 2: Table S-1).

## Abbreviations

CWD: coarse woody debris
IPCC: International Panel on Climate Change
IWD: initial wood density
*k*: rate-constant of decomposition
MAT: mean annual temperature
Q10: quotient 10 or rate of increase with a 10 ℃ increase in temperature
TAP: total annual precipitation
