# Supplementary material for: Release of coarse woody detritus-related carbon: a synthesis across forest biomes
Source: Carbon Balance Manag. 2020 Jan 15;15:1. doi: 10.1186/s13021-019-0136-6 (PMC7227111; doi:10.1186/s13021-019-0136-6)
Supplement: Supplementary file 1 — Additional file 1. Supplemental text, figures, and table. [file 13021_2019_136_MOESM1_ESM.docx]

**Additional Material**

**Climate Variables**

In our analysis in the main text we used two sources of climatic data: 1) that reported by the article (or in some cases that from the nearest climatic station) and 2) that from WorldClim 2 based on the reported location of the study [45]. When reported, climate represents the conditions that the authors considered characteristic. However, in some cases climate data was not reported and moreover those modeling large regions use geographically distributed climate based on climatic stations that is interpolated using models. We used WorldClim 2 as an example of the latter. To assess how well these data sets corresponded with each other we used linear regression (Proc GLM, [46]).

MAT between the two data sets was highly correlated with an r^2^ of 0.86 (Figure S-1). While individual site MAT’s deviated between the two, the regression had an intercept of 0.307 C and a slope of 1.012. This indicates little overall bias. In contrast, there was a definite bias in TAP, with WorldClim values being generally lower than that reported (Figure S-2). The regression indicated that WorldClim 2 was consistently biased 243 mm lower than that reported with a slope of 1.013. However, examined site by site the main issue is that WorldClim 2 greatly underestimated precipitation in mountainous regions, particularly for the Pacific Northwest, a region where excellent climatic data are available. In these cases TAP was underestimated by up to 1700 mm. This lead to an r^2^ of 0.503, much lower than that for MAT. When WorldClim 2 data is used, the range of MAT is quite similar to that reported by authors; however, in the case of TAP the range is generally more restricted (Figure S-3).


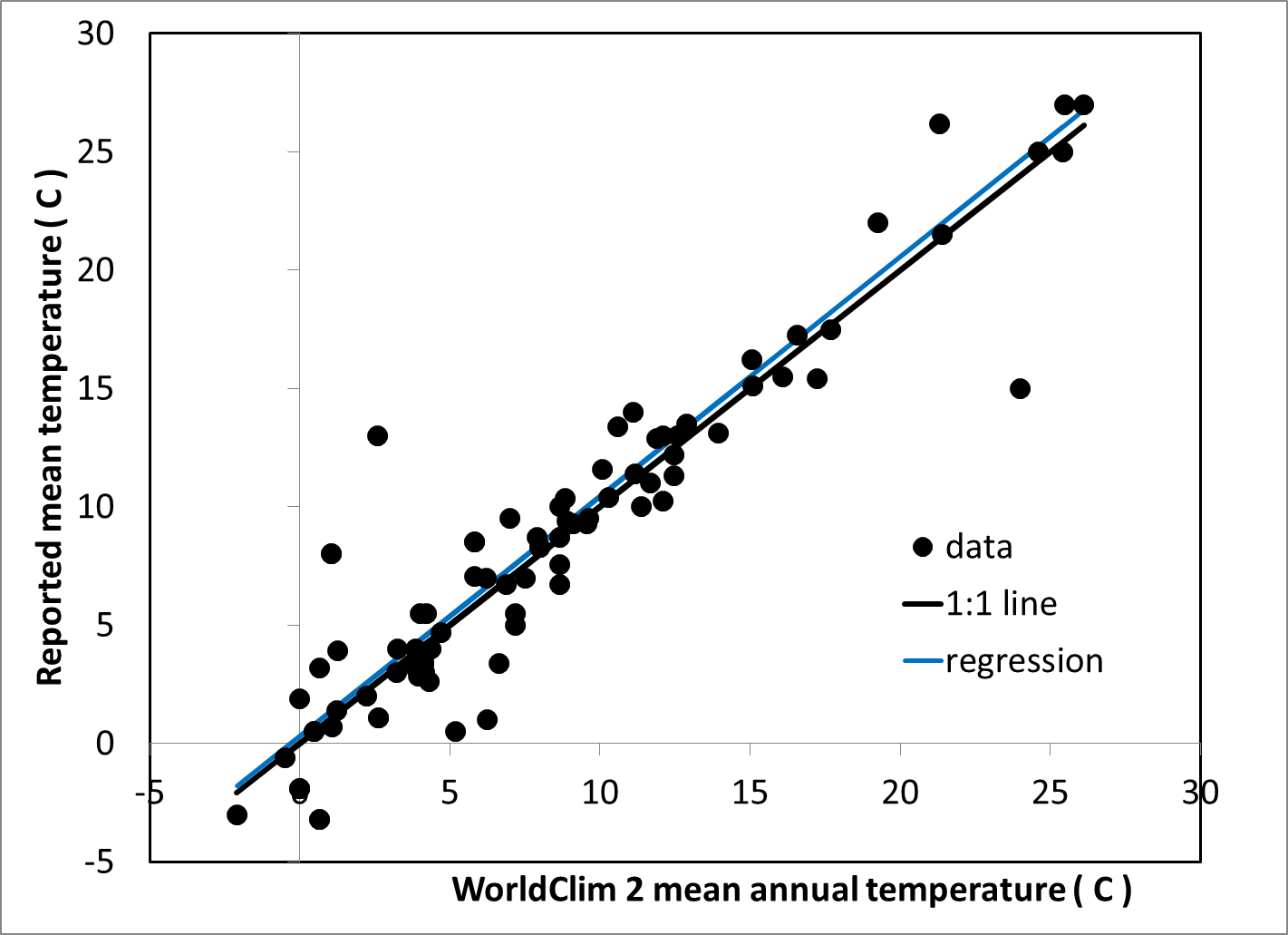


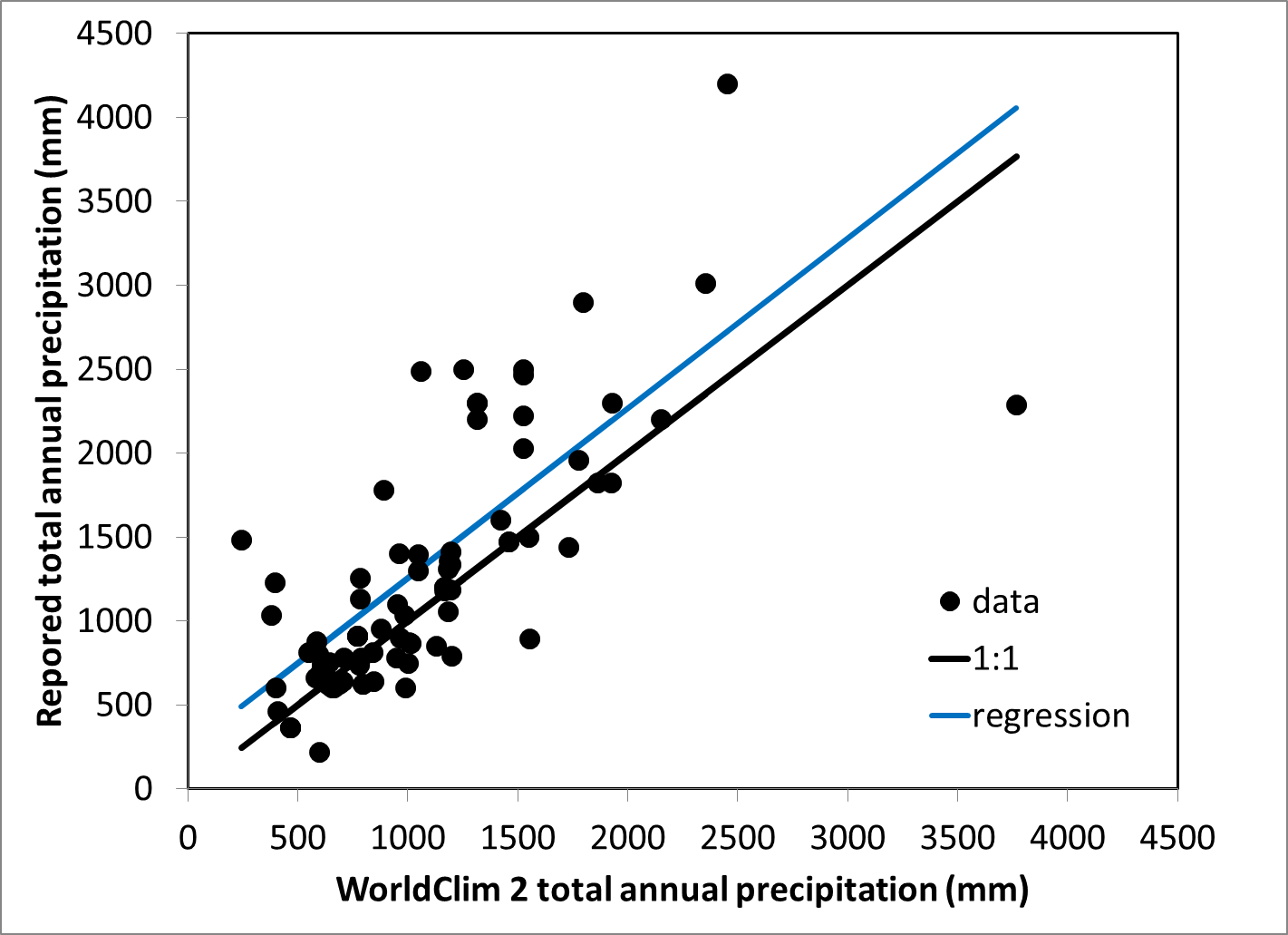
Figure S-1. Relationship between WorldClim 2 mean annual temperature and that reported by a study.

FigureS-2. Relationship between WorldClim 2 total annual precipitation and that reported by a study.

**
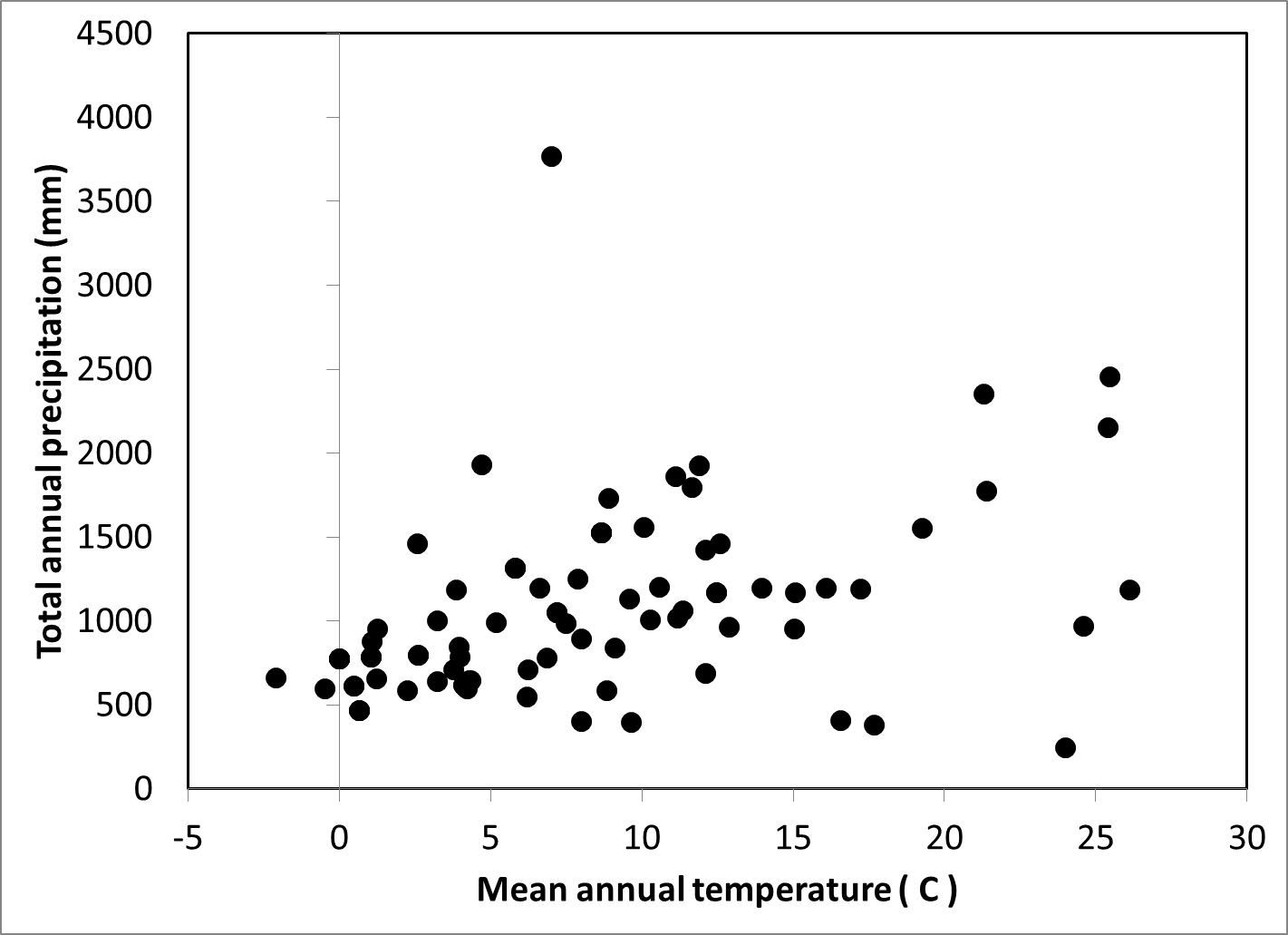
**

Figure S-3. Climatic field of decomposition study sites based on the WorldClim 2 database.

**Model of Decomposition-related Fluxes**

Our model of decomposition-related fluxes following either hurricane or beetle-kill did not account for subsequent production of CWD by the recovering forest. It therefore only examined the effect of the disturbance and CWD that existed just prior to the disturbance. For the hurricane example, we assumed that the decomposition of each species or position could be modeled using a single exponential decomposition function. To determine the decomposition losses for the fastest and slowest species, we assumed that the disturbance created input that was entirely either the most common fastest species or the most common slowest species. For the beetle-kill example we assumed that only *Pinus* died, but that some downed CWD was present at the time of the disturbance. The increase in standing CWD fall rate was modeled using a Chapman-Richards function parameterized so that the snag fall flux reached its highest level after a 10 year lag. Standing CWD losses were comprised of those related to decomposition and fall. The downed CWD pool received inputs from the standing CWD pool and losses via decomposition. Losses from decomposition were calculated as the product of the fraction that would be lost each time step (*i.e.*, *k*) and the store the previous time step. To estimate the effect all the CWD remaining as standing CWD or starting as downed CWD, we reduced the fall rate to zero or increased it to infinitely fast (*i.e*., snags fell immediately), respectively. The decomposition related flux was calculated as the store at time t-1 minus the store at time t.

**Implications of Differences in Decomposition Rate-constants Caused by Differences in Either Size or Species.**

In the review of decomposition rate-constants of CWD (*i.e.*, *k*), there are major differences between sizes and species of CWD. To explore the implications of these findings on modeling the release of carbon following a mortality pulse a set of simulations of hypothetical ecosystems was undertaken. It was assumed there were 10 classes of sizes or species or some combination of the two. The unweighted average decomposition rate-constants for each case were kept at 0.05 per y, but the range of rate-constants within a case was varied so that the ratio of the fastest to slowest rate-constant was 1 (*i.e.*, all the same), 2, 4, 8, or 16. Review of the data indicated that, at least for species, up to a 76-fold difference has been observed between the fastest and slowest decomposing species; therefore this analysis may underestimate the effect of species in some ecosystems. Decomposition rate-constants were assigned to each of the classes so that there was a “steady” rate of change between classes (Figure S-4). Class 1 was assigned the lowest rate-constant, while Class 10 was assigned the highest rate-constant. It was assumed that the disturbance creating tree mortality added 100 MgC/ha at time 0 years. The key variable in determining how important each class was to the overall rate of loss was the proportion that each class contributed to the total input to the forest ecosystem. Five different distributional patterns were assessed: 1) uniform (*i.e.*, all the classes received the same amount of input), 2) a normal distribution in which classes 5 and 6 made the greatest contribution, 3) bimodal distribution in which Classes 1 and 10 made the greatest contribution, 4) a “slow” biased distribution in which Class 1 made the greatest contribution and Class 10 the least, and 5) a “fast” biased distribution in which Class 10 made the greatest contribution and Class 1 the least (Figure S-5).


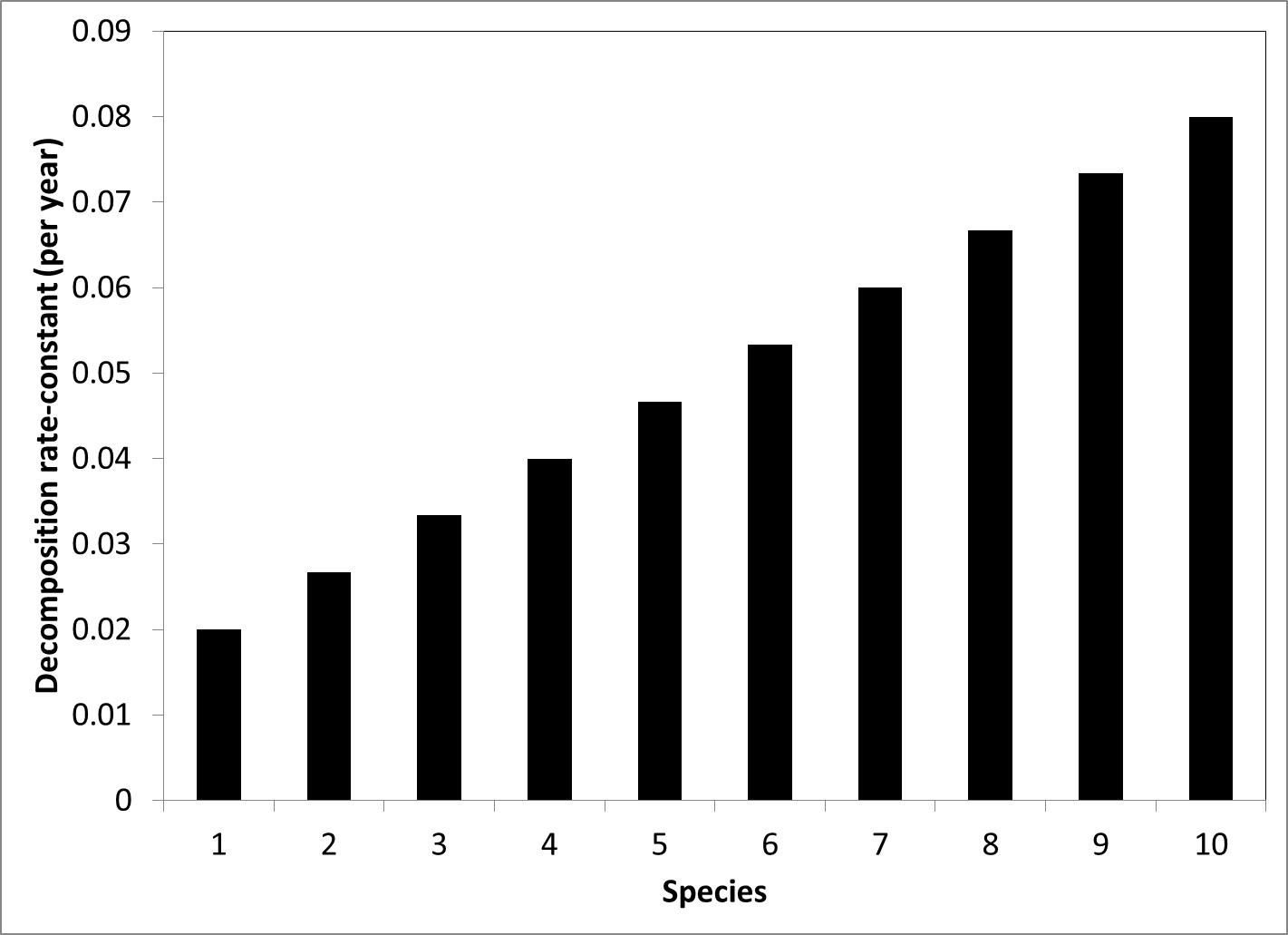


Figure S-4. Example of how the decomposition rate-constant was varied among the 10 species. In this case there was a 4-fold difference between the slowest and fastest decomposing species. The average decomposition rate-constant was 0.05 per y.


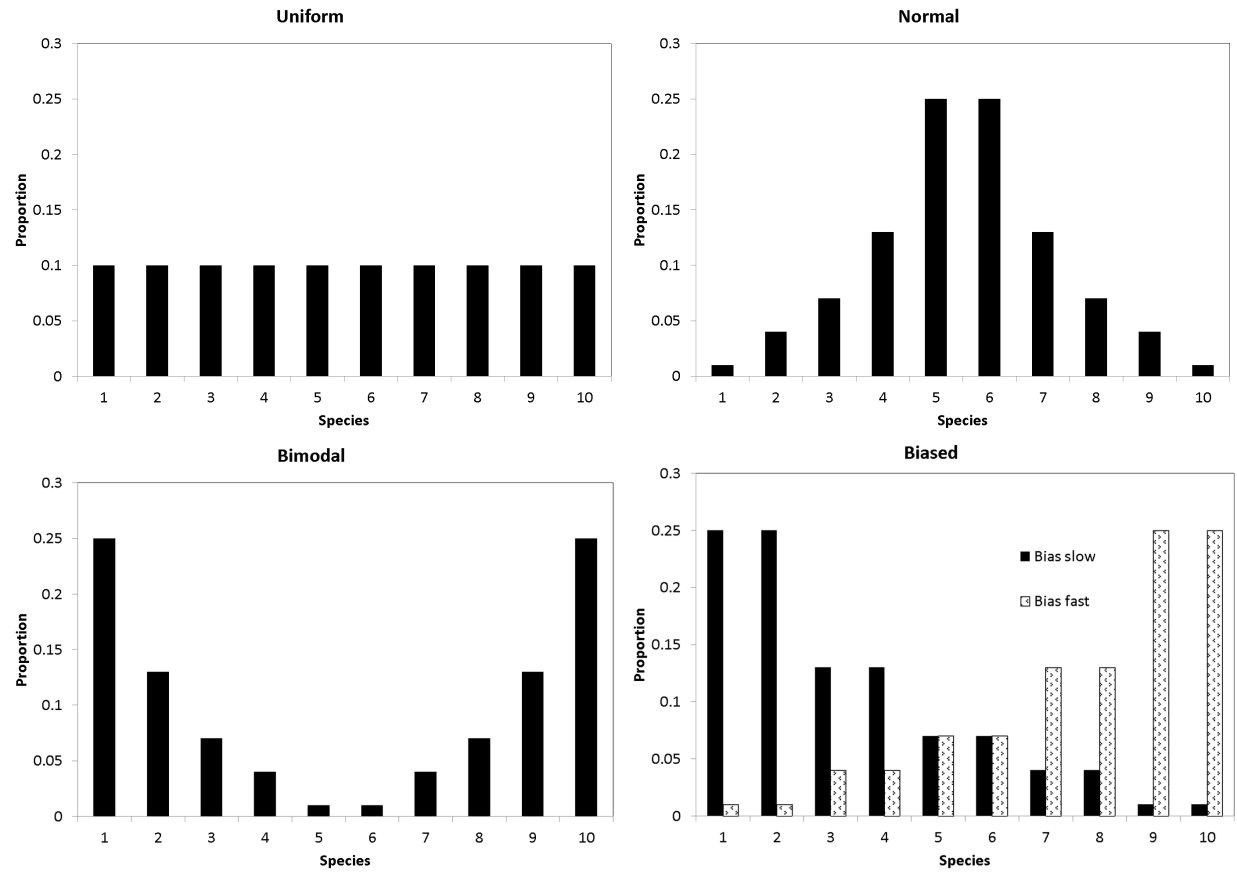


Figure S-5. The distributions assessed to determine the effect of species (or size) abundance on the loss of carbon via decomposition.

The resultant loss fluxes to the atmosphere were plotted to determine the degree that the various ranges in species decomposition rate-constants and distributions influenced the non-linearity of the ecosystem response. If the system behaved in a linear manner it would have the flux temporal pattern of a system in which there was no difference between species (or sizes) and case in which the average decomposition rate-constant was 0.05 per y. While the presence of non-linearity was sometimes suggested by examining the non-transformed data, it was more obvious when the loss flux was logarithmically transformed because linear responses formed straight lines and non-linear ones formed curved lines.

When the decomposition rate-constant was the same for all species, there was no sensitivity to the abundance of species and the first-year loss flux value was 5 MgC/ha/year (Figure S-6). Moreover, the flux declined following a negative exponential curve which appeared as a straight line when logarithmically transformed (Figure S-7). The departure from linearity appeared least for the normal distribution and greatest for the bimodal distribution. Moreover, the degree of non-linearity increased as the ratio between the species with the highest and lowest rate-constants increased. The first-year flux value for the uniform, normal, and bimodal distributions was within 4 % of 5 MgC/ha/year. The largest differences from the cases in which all the species had similar rate-constants occurred after 60 years, suggesting that although the curves are non-linear, the absolute differences in flux values was not of practical significance given much of the pulse of mortality had decomposed by this time (68-96%). The results for biased distributions were considerably different as they were both non-linear and the first-year flux value varied widely from the case in which all species had the same rate-constants. In the case of slow bias, the first-year flux rate was 55% of 5 MgC/ha/year and for fast bias it was 142%. It also appeared that the fast bias case was more non-linear than the slow bias case, with the flux being initially higher, then lower and then eventually higher than the case in which species all decomposed at the same rate. The practical implication of these results is that when species (or size) distributions lead to a weighted average decomposition rate-constant similar to the unweighted average, then it is possible to use the unweighted average of the decomposition-rate constants. However, if there is any slow or fast bias in the distribution of species (or sizes), then a weighted average must be used so that the magnitude and temporal variation in the flux can be modeled accurately. Given the wide possible range in species abundance distributions, it may make the most sense to compute a species weighted average when seeking to simplify by using a single value of a decomposition rate-constants. Although the weighted average evolves over time, this could be simplified by assessing how the abundance of species changes as decomposition proceeds. Moreover, species need not be accounted for *per se* but could be treated as species classes defined by *k*’s. The same could be done for size classes or combinations of size and species classes.


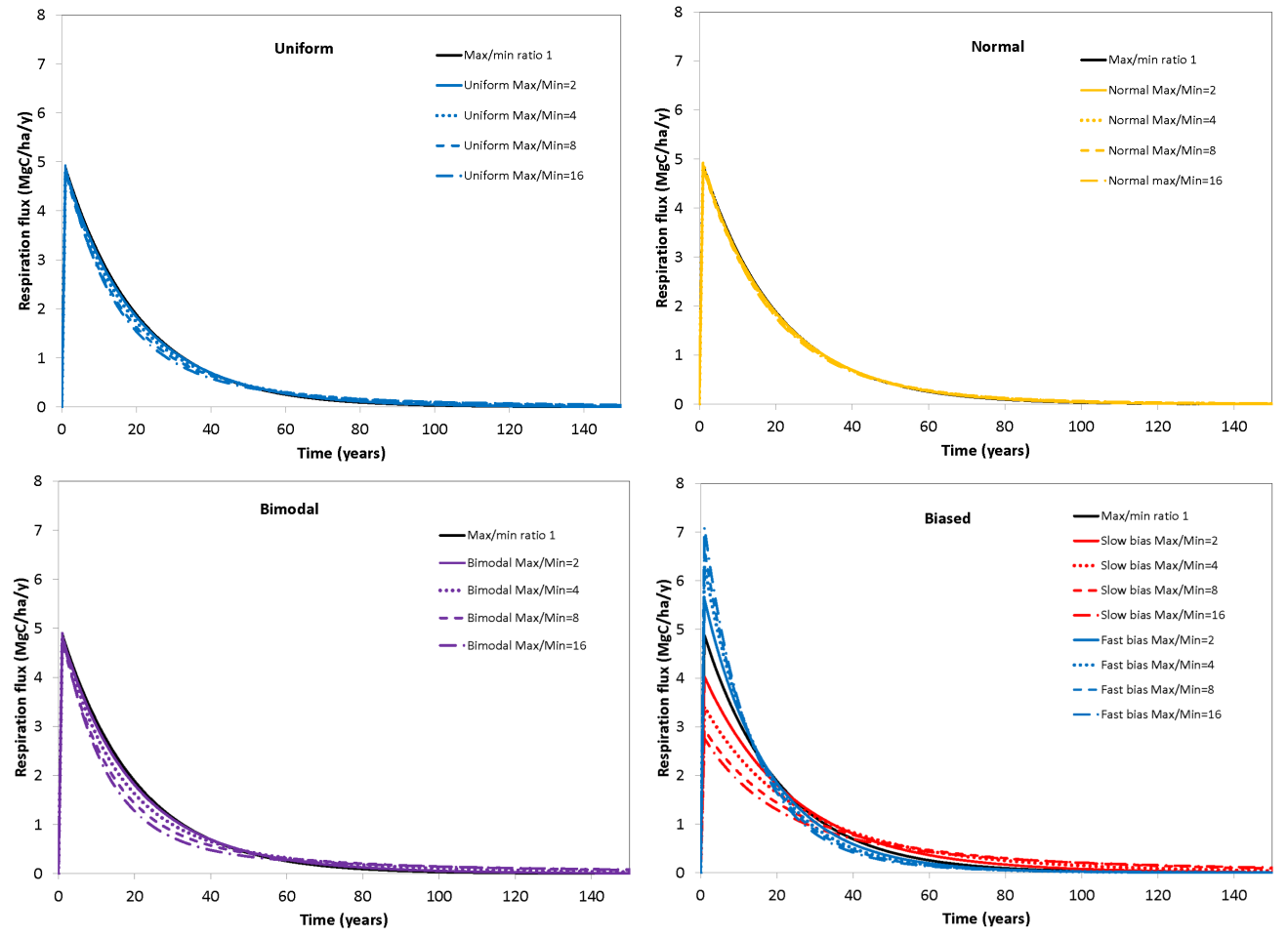


Figure S-6 Non-transformed loss flux response over time for variations in species differences and distributions.


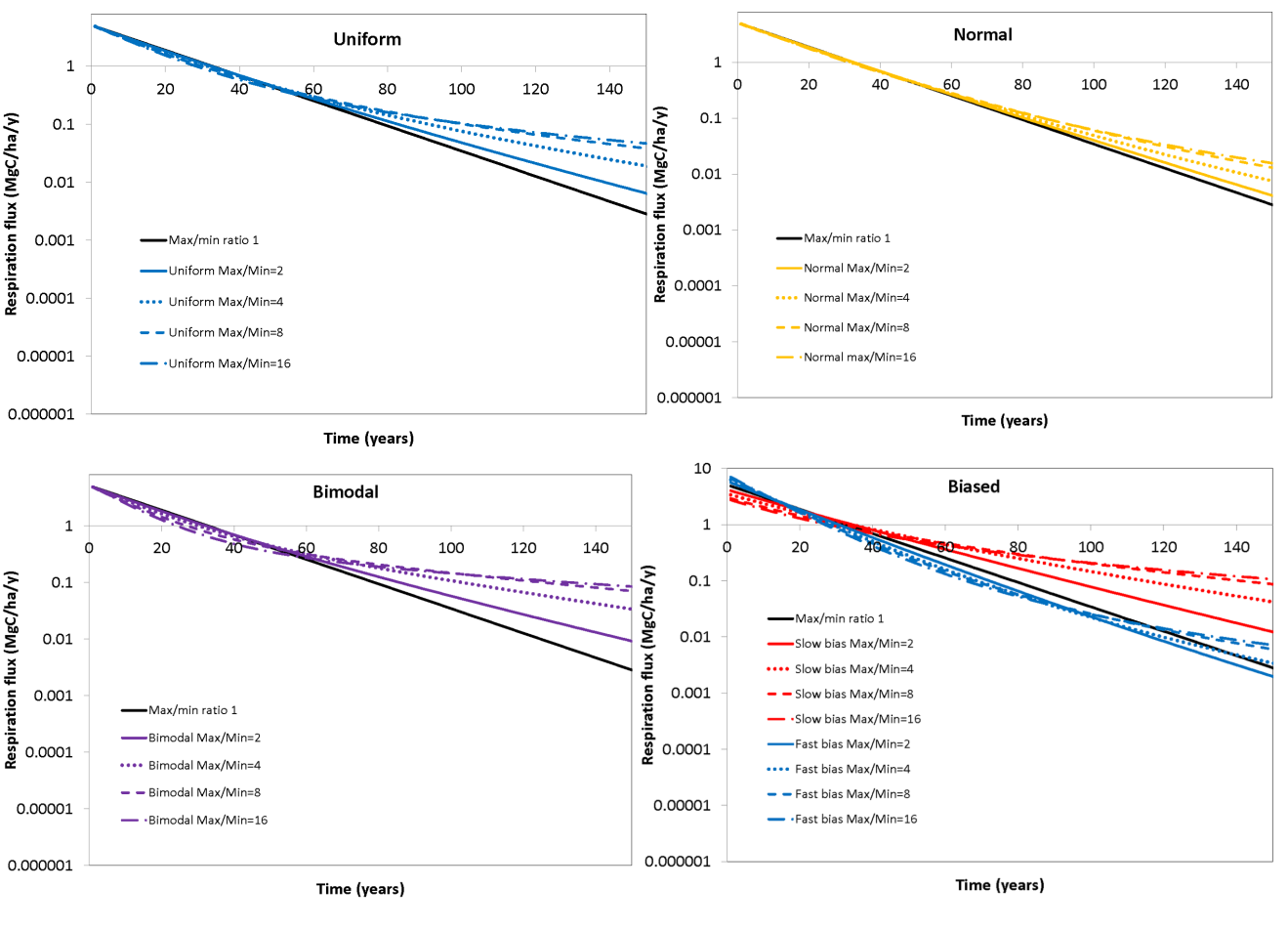


Figure S-7. Logarithmically transformed flux values over time for different ranges of species decomposition rate-constants

**Implications of Differences in Decomposition Rate-constants Caused by Differences in Position**

The review of decomposition-rate constants indicated that there are major differences associated with position of CWD. This implies that as position changes the decomposition rate-constant evolves over time in disturbance-created CWD. The differences between positions are likely caused by differences in microenvironment. The modeling implications of this evolution of *k* were assessed by assuming a disturbance created standing CWD (*i.e.*, snags) that was transformed over time into downed CWD (*i.e.*, logs). Snag fall rate was simulated by assuming snags either started to fall immediately or needed a 10-year period for snag fall to reach its peak. Once they began to fall there were three time frames that were explored for the majority of snags to fall: 1) 15 years, 2) 30 years, and 3) 60 years. It was assumed that the rate-constant of maximum decomposition was 0.05 per y. In one case, representing the situation in mesic to arid climates, it was assumed that snags would decompose slower than logs and the ratio of snag to log decomposition rate-constants was varied from 1, 0.5, 0.25, and 0.125 giving up to an 8-fold difference and a final situation in which snags were assumed to not decompose aboveground (hence the snag *k* was 0 per y). In another case it was assumed that snags would decompose faster than logs. The log to snag decomposition rate-constant ratio was varied between 1, 0.5, 0.25, 0.125, and 0. The latter represents a situation common in boreal forests in which the soil is saturated and mosses quickly over top logs leading to a situation in which logs do not decompose significantly [47].

To describe the response of fluxes to lags three terms were used (Figure S-8). The first was the “ledge” which occurs when there is a lag before snag fall begins. The second is the maximum flux rate and the third is the tail of the flux response.


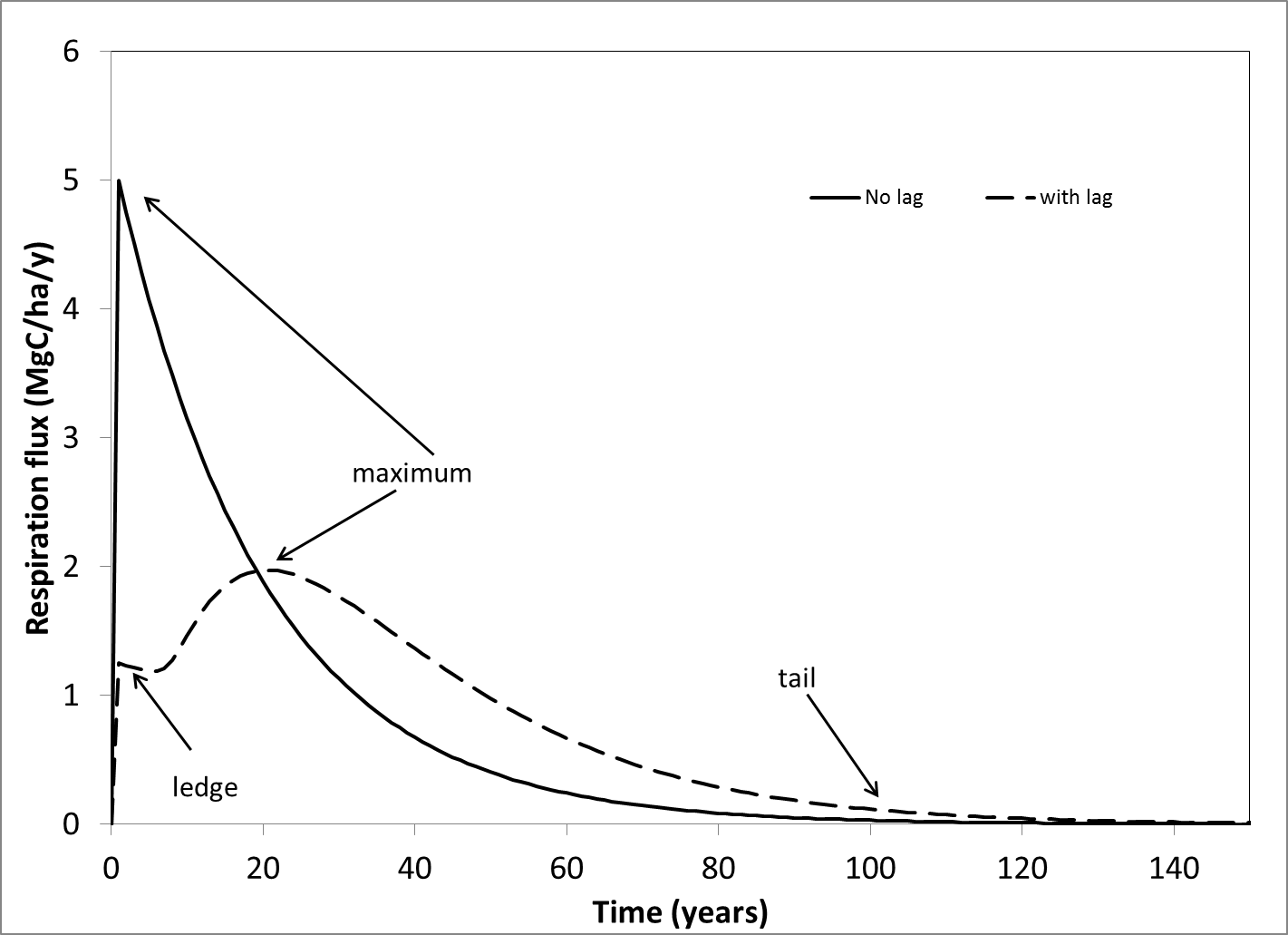


Figure S-8. Terms used to describe the flux of carbon coming from a pulse of mortality caused by disturbance. The ledge occurs when snags decompose more slowly than logs and there is a lag before snags begin to fall. The maximum flux can occur immediately or be delayed. The tail is the latter phase of the flux response.

For the case in which snags decompose more slowly than logs, the effect of having snags gradually fall was to cause the maximum to be lowered and to be delayed relative to the case in which snags and logs decomposed at the same rate (Figure S-9). This was true for all the snag to log decomposition rate-constant ratios examined. The effect of increasing the time for all snags to completely fall was to delay the timing and reduce the value of the maximum flux. When there was a lag before snags began to fall, a ledge appeared in the loss flux rate, and the lower the snag to log decomposition rate-constant ratio, the lower the initial value of the ledge. For example, if the snag to log decomposition rate-constant was 0.5, then the initial value of


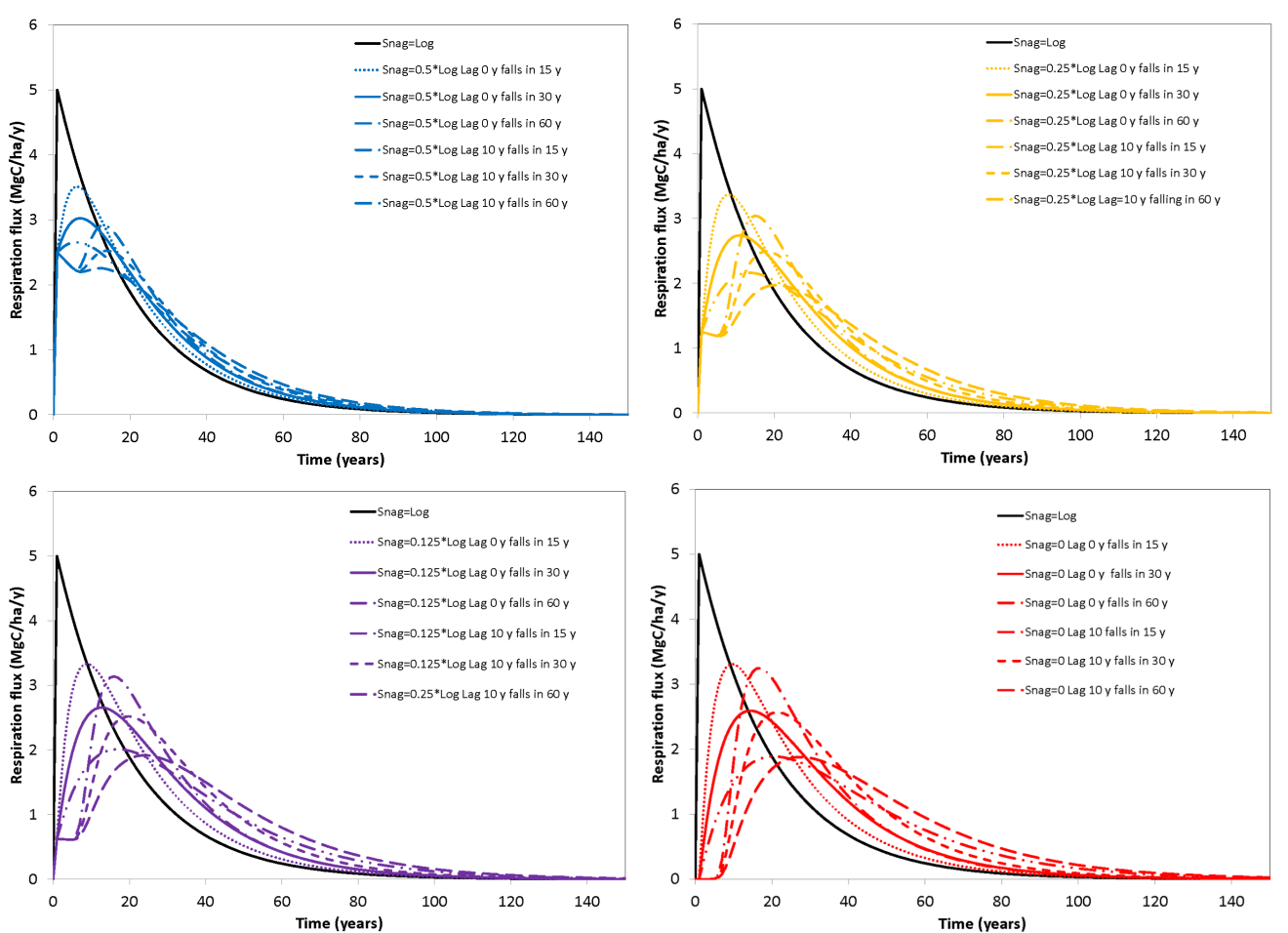


Figure S-9. Flux of carbon emitted caused by disturbance when snags are initially formed and they decompose more slowly than logs by varying degrees.

the ledge was 0.5 the maximum value when this ratio was 1. When this ratio was 0, then the value of the ledge was 0 Mg C/ha/year. The longer the lag time before snags begin to fall and higher the snag to log decomposition rate-constant ratio, the greater the initial decline in the flux and more likely there was a secondary peak in the flux. Of the cases examined this was most evident in when the snag to log decomposition rate-constant ratio was 0.5. However, once the snag to log decomposition rate-constant ratio falls below 0.25, the less likely an initial decline occurs. Finally, when snag fall was gradual, the tail of the flux release from mortality was lengthened; the longer the lag was and the longer it took snags to fall once this process began, the more extended the tail became. It should be noted that even in the case in which snags begin to fall immediately the maximum flux was delayed by 10 years. Moreover, when there was a 10-year lag and then it took an additional 60 years for snags to completely fall the maximum flux was delayed up to 30 years.

In the case in which logs decompose more slowly than snags there is no evidence of a ledge and the maximum flux occurred the first year (Figure S-10). As might be expected, the faster that the snags begin to fall and the faster they completely fall, the faster the overall flux declines. The non-linearity of the flux response increases as the ratio of log to snag decomposition rate-constants increases. This is because, at least for the cases in which this ratio is >0, the flux initially falls below the case when snags and logs decompose at similar rates, but in time it goes above resulting in a long tail of gradual release. An exception is the case in which the log to snag decomposition rate-constant ratio is zero. Because this means that logs do not decompose, it also means that the faster snags fall the faster the overall flux term reaches zero. This results in the potential for substantial amounts of carbon to not be released (at least through the process of decomposition). For example, in the case in which snags begin to fall immediately and are mostly fallen in 15 years, 80% of the disturbance-related mortality would remain unreleased over the long-term. At the other extreme, if there was a 10 year lag before snags began to fall and most had fallen in an additional 60 years, then 35% would remain unreleased over the long-term.

**Conclusions**

These simulation experiments indicate that while it may be possible to address the mixtures of species and sizes with a simplified model using one “average” rate-constant, that this is not possible for a system in which lags occur such as that illustrated by changing of positions. This is because while the former creates non-linear responses in some cases the non-linearity is either relatively minor or its effects appear in stages in which fluxes are low. The latter set of cases involving changes of position creates a highly non-linear response that cannot be mimicked with a single, averaged rate-constant.


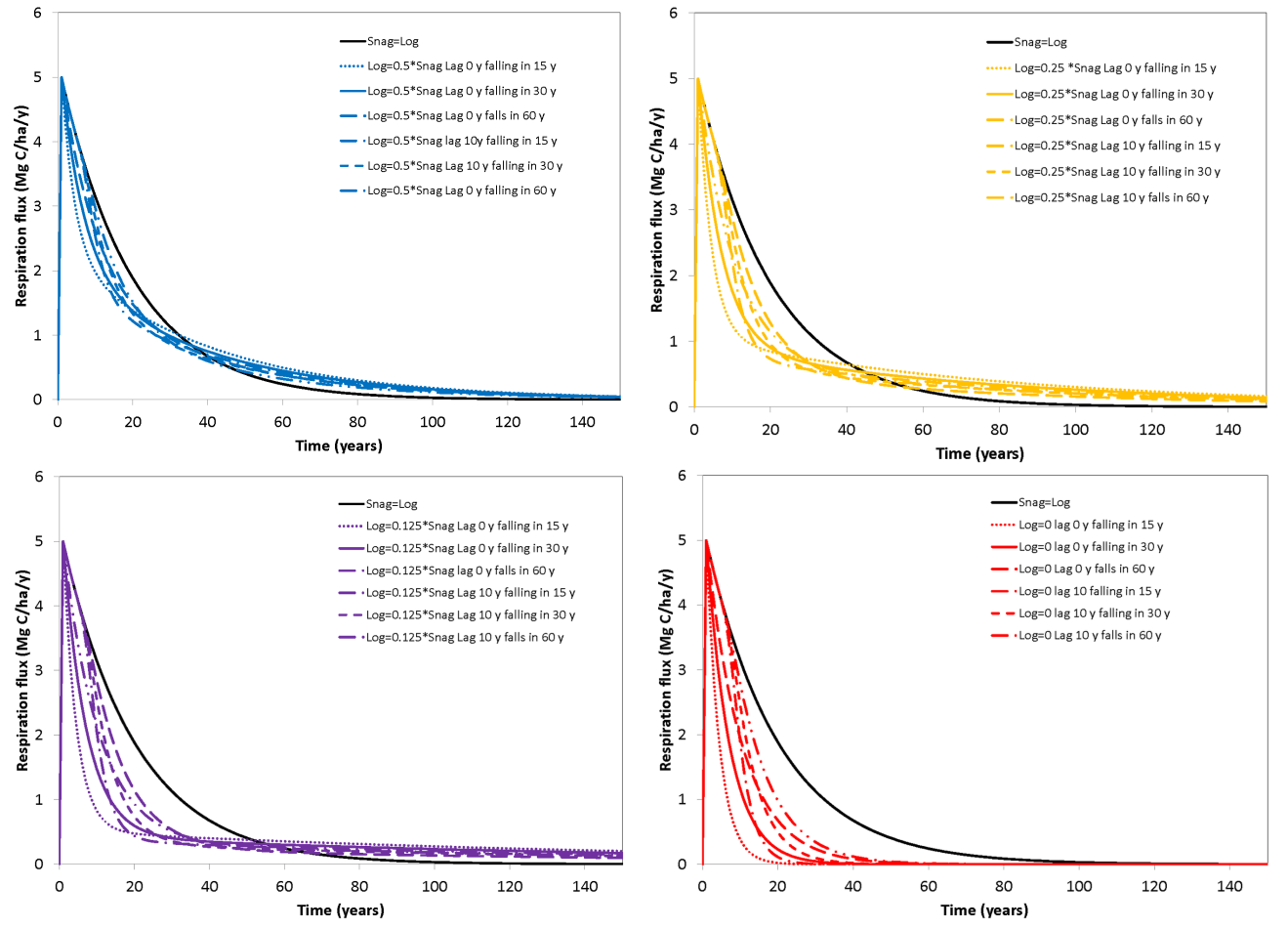


Figure S-10. Flux of carbon emitted caused by disturbance when snags are initially formed and they decompose more quickly than logs by varying degrees.

**Model of Carbon Balance**


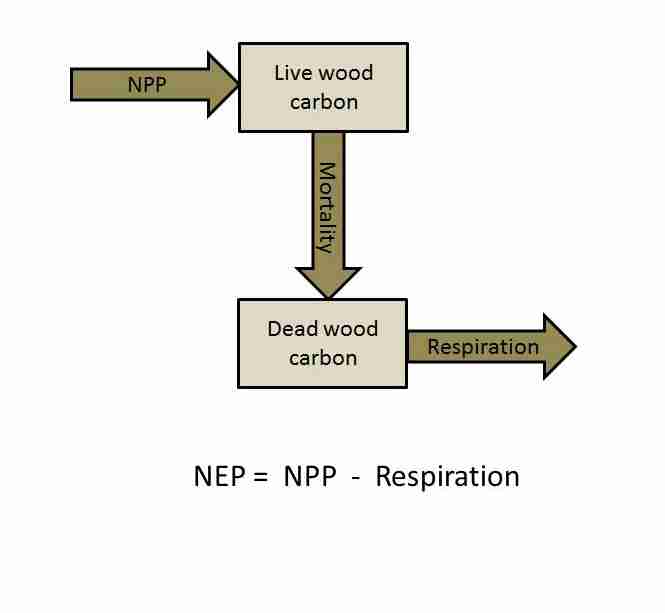


Figure S-11. Flow diagram of carbon used in carbon balance analysis (NEP is net ecosystem production, NPP is net primary productivity).


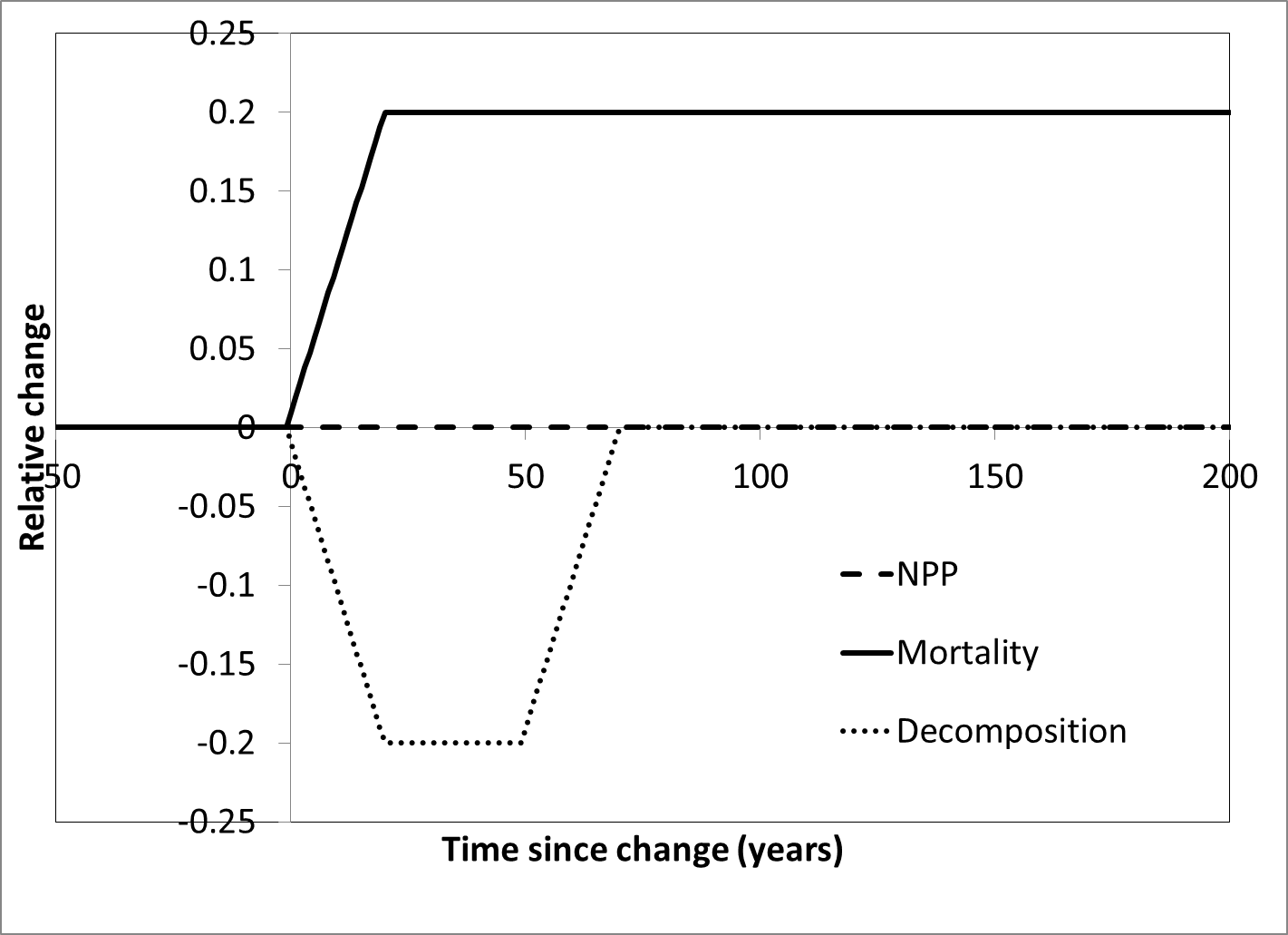


Figure S-12. Example of changes in NPP, mortality, and decomposition after a mortality-pulse starts. In this case mortality was assumed to increase 20% after time 0 and remain high for at least 200 years; decomposition was assumed to decrease 20% for a 50 year period; and NPP was assumed to remain unchanged.

**Modeling Approaches: Description of models**

Table S-2. General aspects of dead wood dynamics and their incorporation in selected examples of forest, vegetation, land use, and/or carbon models.

| Name | Type^1^ | Decomposition^2^ |  |  | Controls^3^ |  |  |  |
| --- | --- | --- | --- | --- | --- | --- | --- | --- |
|  |  |  | Species | Size | Position | Canopy | Climate | Nutrients |
| 4C | FD+M | N | N | N | N | N | Y | Y |
| CBM-CFS3 | VLU | Y | Y^4^ | Y | Y | Y | Y | N |
| EFDM | FD+M | N | NA | NA | NA | NA | NA | NA |
| EFISCEN | FD+M | N | NA | NA | NA | NA | NA | NA |
| FVS | FD+M | Y | N | N | N | N | N | N |
| G4M | FD+M | N | NA | NA | NA | NA | NA | NA |
| GLOBIOM | FD+M | N | NA | NA | NA | NA | NA | NA |
| LPJ-GUESS | DGVM | Y-L | N | Y | N | N | Y | Y |
| YASSO | S | Y-L | N | N | N | N | Y | Y |

1/ FD+M-forest development and management; DGVM-digital global vegetation model; S-soil

2/ Y-yes, CWD considered; Y-L- yes, but only as litter (all dead organic material); N-no

3/ Y-yes; N-no; NA-not applicable

4/ Separates hardwoods and softwoods, but a finer break-down could potentially be included via parameterization
